# Genomic insights into positive selection during barley domestication

**DOI:** 10.1186/s12870-022-03655-0

**Published:** 2022-06-01

**Authors:** Wenjing Tao, Jianxin Bian, Minqiang Tang, Yan Zeng, Ruihan Luo, Qinglin Ke, Tingting Li, Yihan Li, Licao Cui

**Affiliations:** 1 College of Bioscience and Engineering, Jiangxi Agricultural University, Nanchang, Jiangxi, 330045 China; 2 Peking University Institute of Advanced Agricultural Sciences, Weifang, Shandong, 261325 China; 3 College of Forestry, Hainan University, Haikou, Hainan, 570228 China

**Keywords:** Barley, Domestication, Positively selected gene, Gene compactness, Expression pattern, Genetic diversity

## Abstract

**Background:**

Cultivated barley (*Hordeum vulgare*) is widely used in animal feed, beverages, and foods and has become a model crop for molecular evolutionary studies. Few studies have examined the evolutionary fates of different types of genes in barley during the domestication process.

**Results:**

The rates of nonsynonymous substitution (Ka) to synonymous substitution (Ks) were calculated by comparing orthologous genes in different barley groups (wild *vs.* landrace and landrace *vs.* improved cultivar). The rates of evolution, properties, expression patterns, and diversity of positively selected genes (PSGs) and negatively selected genes (NSGs) were compared. PSGs evolved more rapidly, possessed fewer exons, and had lower GC content than NSGs; they were also shorter and had shorter intron, exon, and first exon lengths. Expression levels were lower, the tissue specificity of expression was higher, and codon usage bias was weaker for PSGs than for NSGs. Nucleotide diversity analysis revealed that PSGs have undergone a more severe genetic bottleneck than NSGs. Several candidate PSGs were involved in plant growth and development, which might make them as excellent targets for the molecular breeding of barley.

**Conclusions:**

Our comprehensive analysis of the evolutionary, structural, and functional divergence between PSGs and NSGs in barley provides new insight into the evolutionary trajectory of barley during domestication. Our findings also aid future functional studies of PSGs in barley.

**Supplementary Information:**

The online version contains supplementary material available at 10.1186/s12870-022-03655-0.

## Background

Cultivated barley (*Hordeum vulgare*) is one of the most economically important cereal crops, and its global production value is exceeded only by maize (*Zea maize*), rice (*Oryza sativa*), and wheat (*Triticum aestivum*) [1]. Approximately 75% of the barley produced worldwide is used for animal feed, 20% is used as the primary material in the malting and brewing industries, and the rest (5%) is consumed by humans [2]. Barley has become a key component of the modern human diet, and long-term consumption of barley can substantially reduce the risk of numerous chronic diseases, such as diabetes, cancer, cardiovascular disease, and various inflammatory disorders [3, 4].

The domestication of cultivated barley, which occurred approximately 10,000 years ago in the Near East region referred to as the “Fertile Crescent,” is the process in which wild barley (*Hordeum spontaneum*) was converted into a domesticated crop through artificial selection [5–8]. An increasing number of studies have indicated that barley was domesticated in other regions, such as the Horn of Africa, Morocco, and the Tibetan Plateau [9–11]. The domestication of barley has resulted in a suite of morphological and physiological changes, which are collectively referred to as “domestication syndrome”. These changes affect grain shattering [12], the morphotype of the caryopsis [13], and spike morphology, including fertility of the lateral spikelet in six-row cultivars [14, 15]. Plant domestication is not a single, rapid event but rather a complex gradual process in which target traits are improved by plant breeders through artificial selection [16].

Most domesticated plants have experienced “domestication bottlenecks” in which substantial genetic diversity from their wild ancestors is lost [17]. This bottleneck is a consequence of the limited pool of wild ancestral plants, and it affects the entire genome [18, 19]. Several genes in the genomes of domesticated plants show evidence of previous positive selection [18, 20]*.* Highly favorable alleles experiencing strong positive selection are fixed rapidly, which results in selective sweep signatures in which variation in neighboring genomic regions is eliminated or reduced [21, 22].

The development of high-throughput sequencing technologies has motivated a renewed interest in exploring the targets of positive selection. Signatures of positive selection can be used to identify functionally important genomic regions [23]. Identifying selection signatures will enhance our understanding of the roles of selection and drift in evolutionary processes [24]. Several genome-wide scans for positive selection have been conducted in various species [25–28]. One of the most important statistical methods used to identify deviations from neutrality is the nonsynonymous substitution/synonymous substitution rate (Ka/Ks). The Ka/Ks ratio provides information on the selection pressures operating on a particular gene. Genes are categorized into different types by comparing their Ka/Ks ratios. Ka/Ks > 1 for positively selected genes (PSGs) and Ka/Ks < 1 for negatively selected genes (NSGs), which suggests that these genes have experienced functional constraints, such as deleterious nonsynonymous amino acid substitutions; and Ka/Ks = 1 for neutral genes [29, 30].

This study aimed to identify the PSGs of barley at various stages of its evolution during domestication. Using the barley Morex V2 and the pan-genome [31], we initially identified 18,508 single-copy genes and estimated the Ka, Ks, and Ka/Ks values for each gene pair between wild barley and the landrace (referred to as the domestication process) and between the landrace and the improved cultivar (referred to as the improvement process). We also compared the evolutionary rate, properties, expression patterns, and genetic variation of PSGs and NSGs. Our study revealed several functionally important PSGs, and these candidate genes will provide targets for subsequent functional investigations in barley as well as in other cereal crops.

## Results

### Syntenic relationships of the orthologous genes in barley

Totals of 23,392 and 20,262 single-copy orthologous gene pairs were obtained by OrthoFinder and OrthoMCL, respectively. After cross-validation, 18,508 high-confidence single-copy orthologs remained, accounting for ~ 41.53%, ~ 40.40%, and 56.45% of the genome of wild barley, the landrace barley, and the improved cultivar. Genomic synteny refers to the order of conserved blocks of genes within the chromosomes of two related species [32, 33]. Seventeen syntenic blocks were identified between wild barley and landrace barley, and 10 between landrace barley and the improved cultivar (Fig. S1, Table S1). Syntenic relationships were observed for more than 98% of the single-copy orthologous genes, suggesting that orthologous pairs in barley were highly syntenic across the whole genome. Furthermore, no significant correlation was observed between the number of syntenic genes and chromosome length (one-sided Spearman’s rank correlation, ρ = 0.61, *P* = 0.0833), demonstrating that longer chromosomes did not possess more syntenic genes.

### The distribution of Ka, Ks, and Ka/Ks, as well as their correlations in barley

**Fig. 1:**
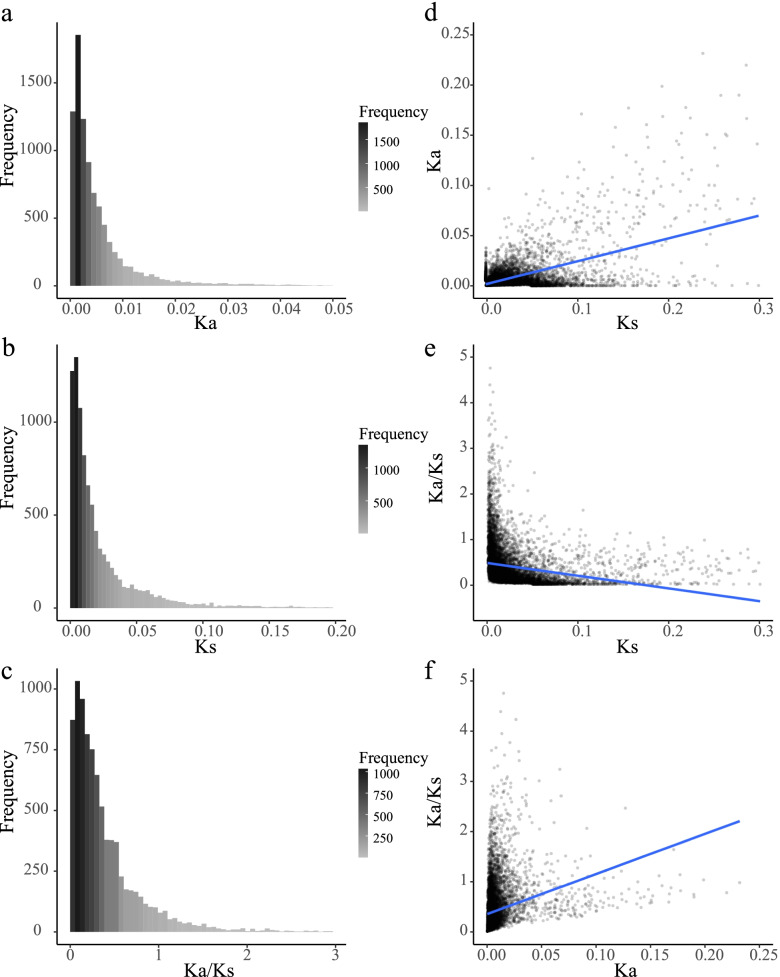
Frequency distributions and correlation analysis of Ka, Ks and Ka/Ks between wild barley and landrace. **a-c** The frequency shows of Ka, Ks and Ka/Ks, respectively. **d** The correlation between Ks (x-axis) and Ka. **e** The correlation between Ks (x-axis) and Ka/Ks. **f** The correlation between Ka (x-axis) and Ka/Ks

The Ka, Ks, and Ka/Ks values were calculated to evaluate constraints on the evolutionary rates of genes. During the initial domestication process, most (80%) Ka values between wild barley and landrace barley ranged from 0.0009 to 0.0138 with an average of 0.0070, whereas the average values were 0.0242 (range 0.0022–0.0589) and 6.3355 (range 0.0576–1.2893) for Ks and Ka/Ks, respectively (Fig. 1, Table S2). Similar results were obtained between the landrace barley and the improved cultivar (Fig. S2, Table S2). The Ka (0.0285) and Ks (0.1473) values in barley were significantly higher than those in *Arabidopsis*, whereas the Ka/Ks value (0.2026) in barley was significantly lower than that in *Arabidopsis* (one-sided Mann–Whitney *U*-test, *P* < 2.20 × 10^–16^, Table S2) [25]. These results suggest that the genes in barley experienced stronger selective pressure than those in *Arabidopsis*. One plausible explanation is that barley is an agriculturally important crop, and higher mutation rates in the selected regions are not allowed and will be discarded by breeders during artificial selection. In contrast, *Arabidopsis* is grown under natural conditions, which facilitates the preservation of a greater number of mutations compared to barley.

**Fig. 2:**
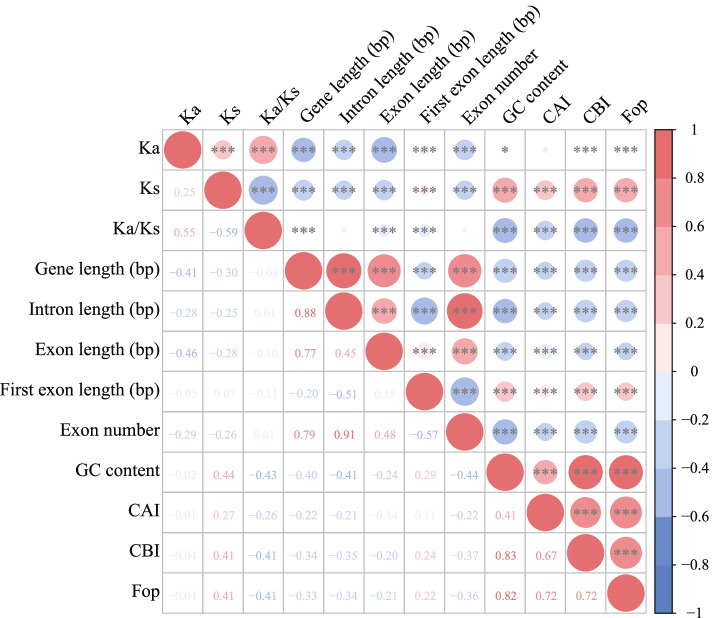
Correlations among substitution rates, gene features and codon usage bias between wild barley and landrace. Upper Right: the size of the circle represents the magnitude of the correlation coefficient, red indicates positive correlation, and blue indicates negative correlation. One asterisk (*), double asterisk (**) and triple asterisk (***) indicate 0.05, 0.01 and 0.001 significant difference level, respectively. Bottom Left: correlation coefficients are presented as Spearman’s rank correlation test ρ

Spearman’s rank correlation tests were conducted to determine the correlations among these parameters. The Ka values were positively correlated with the Ks values (wild *vs.* landrace: ρ = 0.25, *P* < 2.20 × 10^–16^; landrace *vs.* improved cultivar: ρ = 0.25, *P* < 2.20 × 10^–16^, Figs. 1 and 2, Figs. S2 and S3, Tables S3 and S4), which was consistent with *Arabidopsis* (ρ = 0.21) [25], soybean (ρ = 0.22) [34], and *Brassica* (ρ = 0.14) [26]. These results suggest that the common evolutionary mechanisms affecting synonymous and nonsynonymous sites might be shared in different genomes, although the degree of the correlation was slightly different. We further speculate that the positive correlation between Ka and Ks might result from the combination of natural mutation and selection effects [35, 36]. Furthermore, a significant positive correlation was observed between Ka and Ka/Ks and a negative correlation was detected between Ks and Ka/Ks (Figs. 1 and 2, Figs. S2 and S3, Tables S3 and S4).

**Fig. 3:**
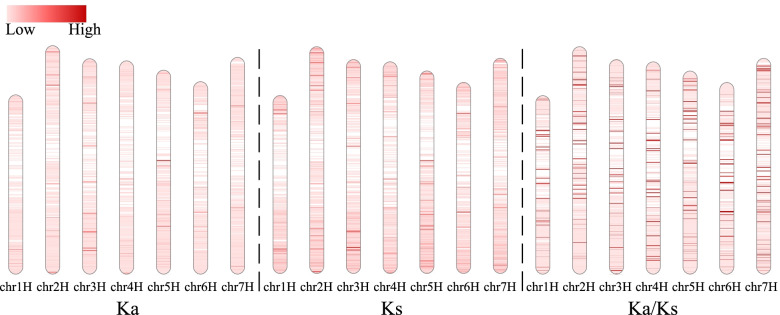
Distributions of Ka, Ks, and Ka/Ks values between wild barley and landrace alongside the chromosome

The genomic distributions of the Ka, Ks, and Ka/Ks values along the chromosomes were determined with a bin size of 50 orthologous genes (Fig. S4). The Ka, Ks, and Ka/Ks values tended to be higher in the distal regions of the centromere than at the centromere (Fig. 3, Figs. S4 and S5). This might be explained by the skewing of meiotic homologous recombination toward the distal ends of the chromosomes in cereals, which facilitates the conservation of sequences concentrated in the centromere [37].

### Identification of PSGs and NSGs in barley

The homologous genes were divided into three categories based on the Ka to Ks ratios: PSGs (Ka/Ks > 1), NSGs (Ka/Ks < 1), and neutral genes (Ka/Ks = 1). Totals of 1,239 and 7,937 domestication-related and 904 and 5,579 improvement-related PSGs and NSGs were identified, respectively (Table S5). In total, 334 genes were under positive selection during the domestication and improvement processes, suggesting that these genes may play key roles in barley and have experienced continuous artificial selection by breeders. Many of the genes could not be categorized because they possessed zero values for Ka, Ks, or Ka and Ks. These genes may reflect a specific type of gene set in the barley genome. Because these genes are subject to strong constraints, they may also experience negative selection (Ka = 0, Ks ≠ 0), positive selection (Ka ≠ 0, Ks = 0), or strong negative selection (Ka = Ks = 0) [26]. These genes were omitted from subsequent analyses.

**Fig. 4:**
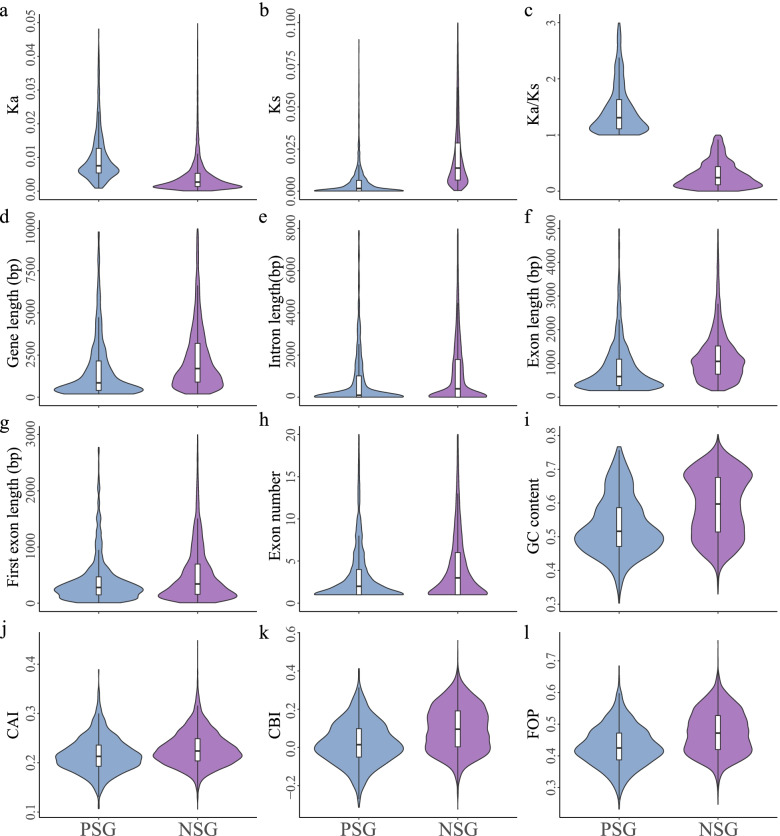
Comparisons of genomic features between PSGs and NSGs during the initial process of barley domestication. The line in the box is the median value, and the lines at the bottom and top of each box are the first (lower) and third (higher) quartiles. Violin plots represent the density of gene numbers. **a-l** The plots display of Ka, Ks, Ka/Ks, gene length, intron length, exon length, first exon length, exon number, GC content, CAI, CBI and Fop between PSGs and NSGs. Blue and purple boxes represent positively and negatively selected genes, respectively

The evolutionary rates between PSGs and NSGs were compared. The Ka and Ks values were unimodally distributed. The Ka value of the PSG peak was higher than that of the NSG peak (Figs. S6a and S7a). In contrast, the Ks values of the PSGs peaked at 0.001, which was lower than that of the NSG peak (0.006) (Figs. S6b and S7b). The average Ka of the PSGs was approximately twice as large as that of the NSGs, and the average Ks of the PSGs was one-fourth less than that of the NSGs (one-sided Mann–Whitney *U*-test, *P* < 2.20 × 10^–16^, Figs. 4 and S8, Table S6). Therefore, the Ka values for PSGs were higher than for NSGs, whereas the Ks values for PSGs were lower than for NSGs.

### Selection mode determines the gene structure during barley evolution

We compared the properties of the genes between PSGs and NSGs to determine how selection shapes gene structure, and similar patterns were observed for the domestication and improvement processes. The density plot revealed a unimodal distribution for the properties of most genes (Figs. S6d-h and S7d-h). The distribution of GC content appeared bimodal (Figs. S6i and S7i). Genes, introns, exons, and first exons were shorter for PSGs than for NSGs (2,051.65 bp *vs.* 2,978.25 bp, 1,177.71 bp *vs.* 1,711.13 bp, 873.94 bp *vs.* 1,267.12 bp, and 405.52 bp *vs.* 519.56 bp, respectively); PSGs also had fewer exons and a lower GC content compared to NSGs (3.30 *vs.* 4.57 and 0.5325 *vs.* 0.5934, respectively) (one-sided Mann–Whitney *U*-test, *P* <  < 0.001, Figs. 4d-h and S8d-h, Table S6).

A correlation analysis was performed between the Ka/Ks values and the gene properties to compare the gene structure between PSGs and NSGs. The Ka/Ks ratio was significantly negatively correlated with gene length and exon length (two-sided Spearman’s rank correlation test, *P* < 0.05, Figs. 2 and S3, Tables S3 and S4), suggesting that the evolutionary rate and selective mode can affect the structure of genes in barley.

### Expression patterns and tissue specificity analysis of PSGs and NSGs

An increasing number of studies have suggested a link between the evolutionary rate and the gene expression pattern [38–41]. The expression pattern of each gene was evaluated using fragments per kilobase of exon per million fragments mapped (FPKM). Because of the limitations of the matched RNA-seq dataset, we only obtained the expression patterns of genes in different tissues/stages for the improved barley cultivar. The overall expression level of PSGs was lower than NSGs. The orthologous genes were subsequently divided into two categories based on criteria from a previous study: highly expressed genes (FPKM ≥ 50) and weakly expressed genes (FPKM ≤ 3) [42]. We detected divergent expression patterns between PSGs and NSGs (one-sided Fisher's exact test, *P* = 1.277 × 10^–11^, Table S7). A total of 36.95% (334 genes) of the PSGs were weakly expressed, and 6.64% (60 genes) were highly expressed. A total of 23.41% (1,306 genes) of the NSGs were weakly expressed and 10.65% (594 genes) were highly expressed.

We next analyzed whether the orthologs with different evolutionary rates exhibited tissue specificity. The orthologs were classified into two groups based on the type of tissue specificity: categorical tissue specificity (expressed in only one tissue, also defined as τ = 1) and overall tissue specificity (expressed in two or more tissues, also defined as τ < 1) [43]. Categorical tissue specificity was more commonly observed among PSGs than NSGs. A one-sided Fisher’s exact test revealed that the tissue specificity of PSGs was significantly higher than that of NSGs (*P* = 0.0118, Table S7). Specifically, nine (1.00%) PSGs were classified as categorical tissue specificity genes, and 607 (67.15%) were classified as overall tissue specificity genes. In contrast, only 23 (0.41%) NSGs were categorical tissue specificity genes, and 4,410 (79.05%) were overall tissue specificity genes. Next, we performed a correlation analysis between the evolutionary mode and gene expression. The data showed that the Ka/Ks ratio was negatively correlated with the expression level (ρ = –0.10, *P* = 3.66 × 10^–16^, Fig. S3, Table S4). However, the Ka/Ks ratio was positively correlated with tissue specificity (ρ = 0.04, *P* = 0.0027, Fig. S3, Table S4).

### Comparison of codon usage bias in PSGs and NSGs

Codon usage bias is usually defined as the species-specific deviation from the uniform usage of codons during translation from genes to proteins [44]. Selection for translational accuracy is thought to be the driver modulating codon usage bias in various species [45, 46]. Various codon usage indicators, such as the codon adaptation index (CAI), codon bias index (CBI), and frequency of optimal codons (Fop), were calculated for PSGs and NSGs to clarify how selection has shaped the evolution of codon usage bias in barley. The overall average CAI, CBI, and Fop values were 0.2273, 0.0898, and 0.4694 for landrace barley, respectively. The average CAI was 0.2271, the average CBI was 0.0907, and the average Fop was 0.4698 for improved barley cultivar, indicating weak usage bias across the barley genome (Table S8). Furthermore, correlation analysis showed that Ka/Ks was negatively associated with CAI (wild *vs.* landrace: –0.26; landrace *vs.* improved cultivar: –0.24), CBI (wild *vs.* landrace: –0.41; landrace *vs.* improved cultivar: –0.40), and Fop (wild *vs.* landrace: –0.41; landrace *vs.* improved cultivar: –0.40), and these differences were all significant (two-sided Spearman’s rank correlation test, *P* < 2.20 × 10^–16^, Figs. 2 and S3, Tables S3 and S4). Significantly lower CAI, CBI, and Fop values were observed for PSGs compared to NSGs (Mann–Whitney *U*-test, *P* <  < 0.001, Table S6). These results suggest that selection might make a greater contribution to shaping patterns of codon usage bias in barley compared to mutation.

Correlation analysis was carried out to evaluate the effect of codon usage bias on gene composition. The three codon bias parameters (i.e., CAI, CBI, and Fop) were negatively correlated with gene length, intron length, exon length, and exon number (two-sided Spearman’s rank correlation test, *P* <  < 0.001, Figs. 2 and S3, Tables S3 and S4). Expression levels were negatively correlated with CAI, CBI, and Fop (two-sided Spearman’s rank correlation test, *P* < 0.001), whereas tissue specificity was positively correlated with CAI (two-sided Spearman’s rank correlation test, *P* = 0.0359, Fig. S3, Table S4).

### Transcription factor (TF) identification and PSG enrichment analysis

**Fig. 5:**
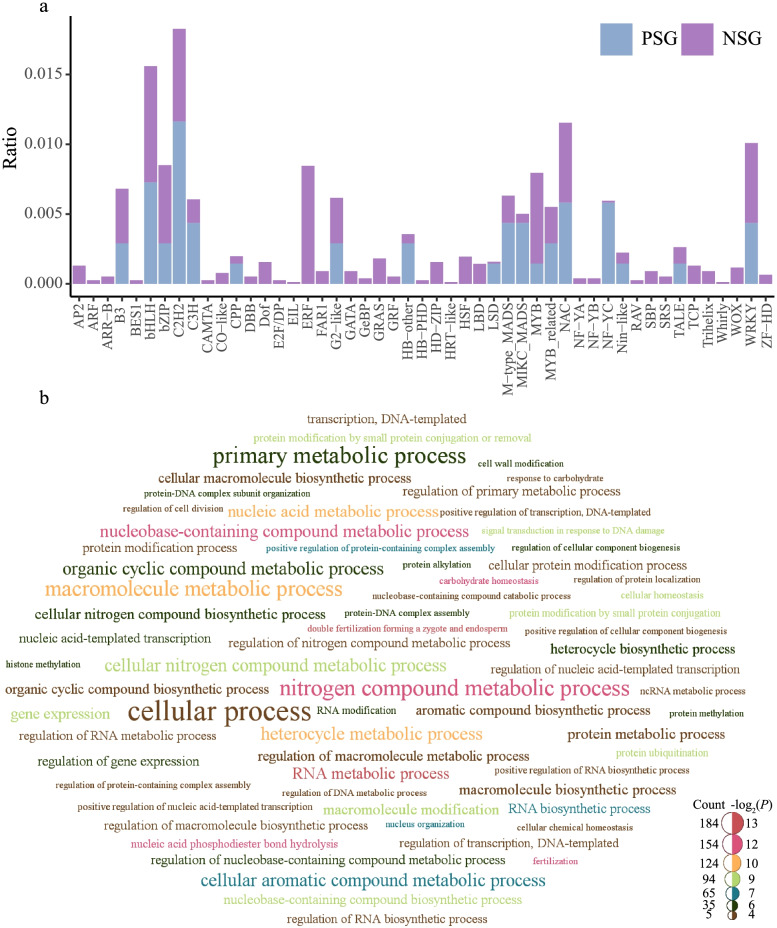
Identification of TFs and functional enrichment analysis of PSGs between wild barley and landrace. **a** Frequency distributions of transcription factors. Blue and purple columns represent positively and negatively selective genes, respectively. **b** Word clouds of GO terms of PSGs. The larger word of the term indicates gene numbers that were more enriched. Terms with *P* < 0.05 were selected

One major focus of our analyses was the positively selected TFs. TFs possess similar functional domains and perform many physiological and metabolic functions by binding to promoter and enhancer regions [47]. TF gene families are divided into different subgroups or subfamilies based on the sequence composition of the core domain. A total of 1,582 one-to-one orthologous groups were characterized as TFs (Table S9). Forty-eight PSGs and 667 NSGs were identified as TFs for the wild *vs.* landrace comparison, and 41 PSGs and 451 NSGs were identified as TFs for the landrace *vs.* improved cultivar comparison (Fig. 5a and S9a, Table S10). Various functional TFs, such as bHLH, C2H2, NAC, and B3, were primarily encoded by PSGs (Tables S10 and S11), suggesting that these genes experienced strong artificial selection and thus could provide targets for domestication and improvement.

Gene Ontology (GO) and Kyoto Encyclopedia of Genes and Genomes (KEGG) enrichment analyses were performed to characterize the biological functions of the PSGs. During the wild barley domestication process, 15, 9, and 67 GO terms were significantly enriched in cellular components (CC), molecular functions (MF), and biological process (BP), respectively (Fig. S10, Table S12). The PSGs were highly enriched in RNA metabolic process (GO:0016070), fertilization (GO:0009566), carbohydrate homeostasis (GO:0033500), nitrogen compound metabolic process (GO:0006807), and nucleic acid metabolic process (GO:0090304) (Fig. 5b, Table S12). The enrichment analysis of the KEGG pathways included protein families: genetic information processing, brite hierarchies, and ubiquitin system (Table S13). During the improvement of landrace barley, we identified 19 enriched GO terms in CC, 5 enriched GO terms in MF, and 49 enriched GO terms in BP (Fig. S11, Table S12). The significantly enriched GO terms included cellular nitrogen compound metabolic process (GO:0034641), metabolic process (GO:0008152), negative regulation of developmental process (GO:0051093), and floral organ development (GO:0048437) (Fig. S9b, Table S12). Moreover, 14 KEGG pathways were significantly enriched (Table S13).

### PSGs exhibited greater losses of genetic diversity than NSGs during barley domestication

Genome-wide single nucleotide polymorphism (SNP) analysis has been used to characterize the genetic diversity of barley during domestication [48]. Using whole exome-captured resequencing data, we obtained 255,364 high-confidence SNPs. Most of the SNPs were located in intron regions (60.31%), followed by synonymous variants (19.98%) and nonsynonymous variants (19.71%), as the reading frame-independent variants were under weaker negative selection than the frame-change variants (Table S14).

**Fig. 6:**
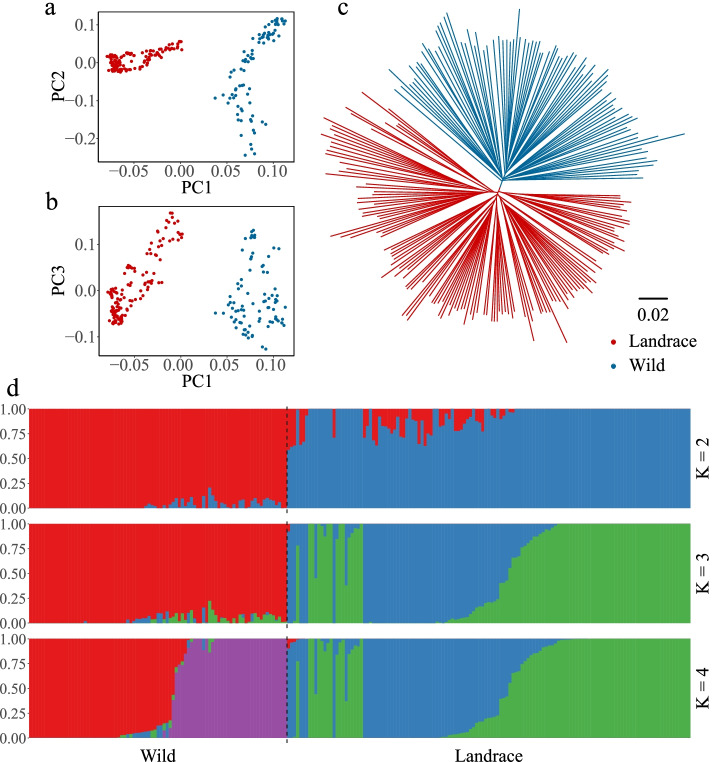
Population structure of wild barley and landrace accessions based on PSG-related and NSG-related SNPs. **a** Principal component analysis PC1 *vs.* PC2. **b** Principal component analysis PC1 *vs.* PC3. **c** The NJ phylogenetic tree. **d** Population structure with K ranging from 2 to 4

We next characterized the genetic divergence and evolutionary history of the wild and landrace barley populations through phylogenetic analysis. The phylogenetic tree revealed two genetically divergent populations corresponding to the landrace and wild barley rather than barley populations with different geographic origins (Fig. 6c). The results of the principal component analysis (PCA) were consistent with the phylogenetic relationships. The first principal component explained 8.07% of the total variance and captured the biological differentiation between landrace and wild barley. The second and third principal components were correlated with the geographical origins of barley and explained 4.31% and 3.91% of the total variance, respectively (Fig. 6a and b, Table S15). The results of the admixture analysis and PCA were consistent. When K = 2, the two groups coincided with landrace and wild barley (Figs. 6d, S12d, S13d). These results suggest that artificial selection has played a major role driving the divergence between wild barley and landrace barley.

**Fig. 7:**
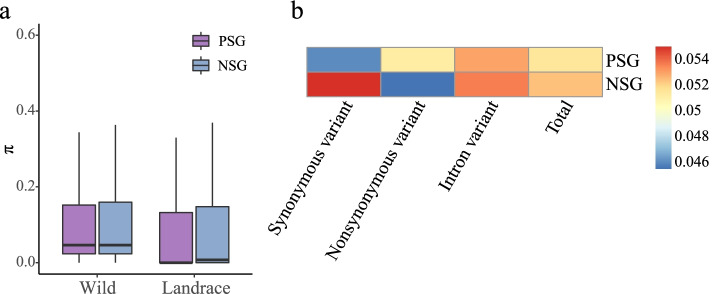
Population divergence between wild barley and landrace. **a** Distributions of nucleotide diversity (π). **b** *F*_*ST*_ within different genomic regions

Nucleotide diversity (π) is a measure of genetic diversity that provides insight into the evolutionary history of a species [49, 50]. The π values were higher for PSGs and NSGs in wild barley than in landrace barley (PSGs: π_wild_ = 0.1147 *vs.* π_landrace_ = 0.0952; NSGs: π_wild_ = 0.1186 *vs.* π_landrace_ = 0.1027), indicating that barley underwent a profound genetic bottleneck during domestication. The reduced genetic diversity of PSGs was significantly greater than that of NSGs (PSGs *vs.* NSGs: ~ 17.00% *vs.* ~ 13.41%) (one-sided Mann Whitney *U*-test, *P* < 2.20 × 10^–16^, Fig. 7a, Table S16).

We calculated the *F*_*ST*_ (Wright’s F-statistic) index to evaluate the genetic differentiation between the wild and landrace populations [51]. The *F*_*ST*_ value of PSGs was higher than that of NSGs for nonsynonymous variants (PSGs: *F*_*ST*_ = 0.0514, NSGs: *F*_*ST*_ = 0.0457, Table S17) (Fig. 7b), suggesting that beneficial nonsynonymous mutations under positive selection have been retained and gradually spread in landrace barley via artificial selection.

### Analysis of the orthologs, expression, and haplotypes of the candidate PSGs

**Fig. 8:**
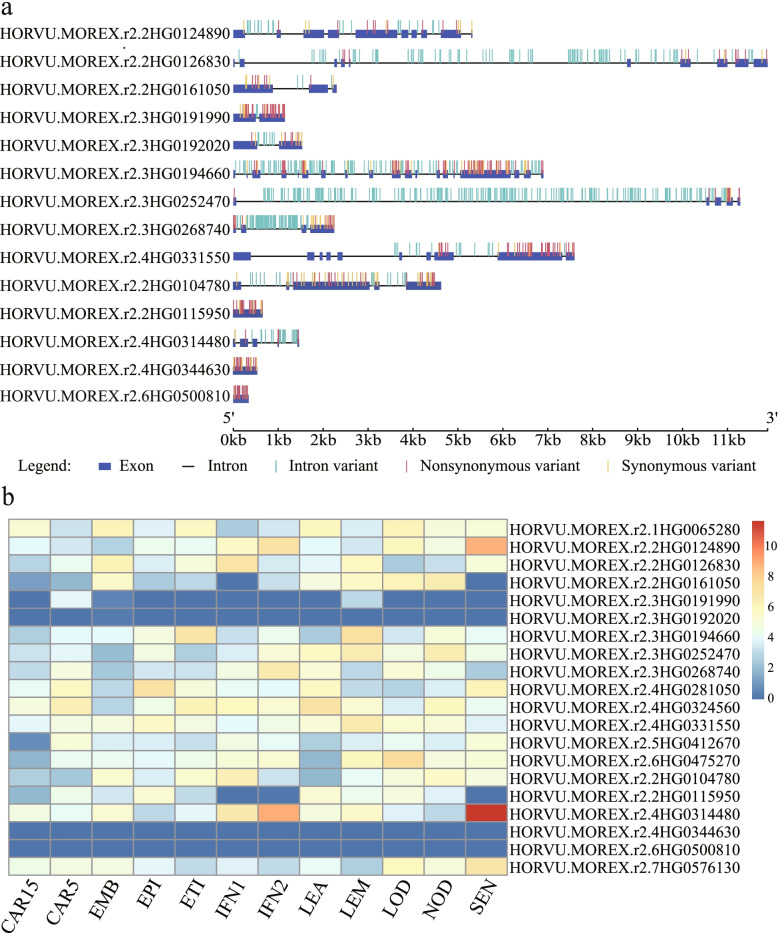
Distributions of nucleotide variants within the candidate PSGs (**a**) and tissues/stages expression profile (**b**). FPKM values were normalized by log_2_(FPKM + 1) transform to represent color scores. CAR15: bracts removed grains at 15DPA; CAR5: bracts removed grains at 5DPA; EMB: embryos dissected from 4 d-old germinating grains; EPI: epidermis with 4 weeks old; ETI: etiolated from 10-day old seedling; INF1: young inflorescences with 5 mm; INF2: young inflorescences with 1–1.5 cm; LEA: shoot with the size of 10 cm from the seedlings; LEM: lemma with 6 weeks after anthesis; LOD: lodicule with 6 weeks after anthesis; NOD: developing tillers at six-leaf stage; SEN: senescing leaf

A BLAST search was conducted against the Ricedata database to probe the biological functions of the PSGs. Twenty highly similar matches with rice were identified for barley PSGs. For example, *HORVU.MOREX.r2.2HG0161050*, *HORVU.MOREX.r2.3HG0191990*, *HORVU.MOREX.r2.3HG0194660*, *HORVU.MOREX.r2.4HG0324560*, and *HORVU.MOREX.r2.4HG0314480* were orthologous to *ETA1, OsSWN3*, *HEIP1*, *GLUP6*, and *DEP2* in rice, respectively. We further profiled the spatiotemporal expression levels in 12 development stages/tissues. A total of 18 genes (except for *HORVU.MOREX.r2.4HG0344630* and *HORVU.MOREX.r2.6HG0500810*), with an average τ value of 0.7947, exhibited high tissue specificity (Fig. 8, Table S18). Among them, *HORVU.MOREX.r2.3HG0194660* showed relatively high expression in the lemma; *HORVU.MOREX.r2.6HG0475270* was predominantly expressed in the lodicule; *HORVU.MOREX.r2.2HG0126830* and *HORVU.MOREX.r2.3HG0268740* were highly expressed in inflorescences; and *HORVU.MOREX.r2.1HG0065280* was differentially expressed in the embryo and lodicule.

**Fig. 9:**
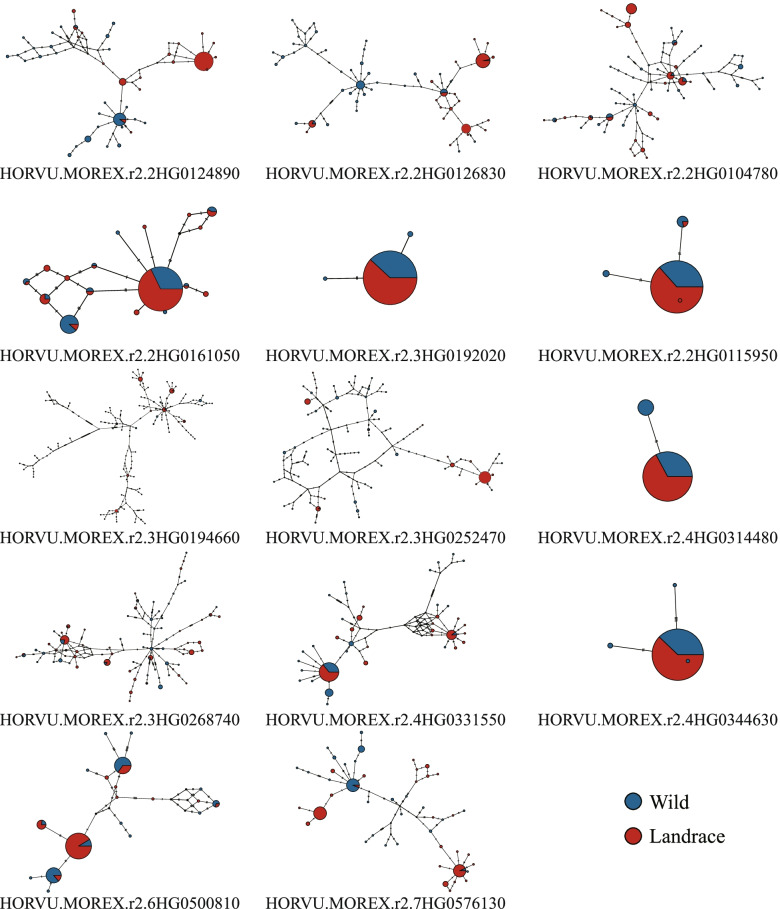
Median-Joining haplotype networks of candidate genes in wild barley and landrace populations. The circle size represents the number of accessions holding a particular haplotype. The blue and red circles refer to wild barley and landrace accessions, respectively

A haplotype consists of closely linked genetic variants that tend to be inherited together [52]. We constructed the haplotype networks for these candidate PSGs using SNPs. Because rare alleles were filtered out, the haplotype networks were not obtained for six PSGs. A total of 549 haplotypes (362 haplotypes for wild barley, and 238 for landrace barley) were identified for the remaining 14 PSGs (Fig. 9, Table S18). A clear pattern of differentiation was detected between the wild barley and landrace accessions. Differences between the wild-specific or landrace-specific haplotypes reflected divergence due to artificial selection. Consistent with the large reduction of π in the landrace populations, the abundance of rare haplotypes in wild populations greatly increased haplotype polymorphism, which reflects their high potential for genetic improvement.

## Discussion

### PSGs were less numerous and evolved more rapidly than NSGs during barley domestication

Positive and negative selection are two important types of natural selection [53], and the relative roles they play in shaping the evolution of nuclear genes remain unclear. The most updated reference genome and the pan-genome of barley provide an opportunity to investigate the evolutionary changes that have occurred during the domestication and improvement of barley through comparative genome analysis [31, 54]. We characterized the differences between the PSGs and NSGs of wild barley and landrace barley, as well as between landrace barley and the improved barley cultivar. A total of 18,508 single-copy orthologous genes were identified. Most of the genes were categorized into two types according to the Ka/Ks ratio: PSGs (Ka/Ks > 1) and NSGs (Ka/Ks < 1). A total of 1,239 (wild *vs.* landrace) and 904 (landrace *vs.* improved cultivar) PSGs were identified, accounting for 2.70% and 2.76% of the whole genome, respectively. The lower proportion of PSGs relative to NSGs is consistent with the findings of previous studies [25, 26, 28]. Most mutations have deleterious fitness effects and thus are lost rapidly [55]. These studies have shown that PSGs tend to have higher Ka and lower Ks values compared to NSGs in various species [25, 26, 28]. A similar pattern was observed in our study. The Ka and Ks values were two-fold higher and four-fold lower in PSGs compared with NSGs, respectively. These findings indicate that the evolutionary rate of PSGs was more rapid than that of NSGs.

### Gene compactness, codon usage, and expression profiles of PSGs and NSGs

The relationship between gene expression and gene compactness has been well documented. Highly expressed genes tend to be shorter, possess fewer introns and exons, and have shorter coding regions [56–59]. Woody et al*.* proposed that expression breadth and exon length are positively correlated in genes expressed at low and intermediate levels, but negatively correlated in highly expressed genes [60]. However, the effect of different types of selection on gene structure remains poorly understood. In our study, PSGs experienced strong artificial selection during barley domestication, which altered their structure. PSGs were shorter and possessed shorter introns, exons, and first exons; this finding is consistent with the results of previous studies in *Brassica* [26] and *Pyrus* [28]. Our results suggest that similar modes of selection have operated in various species and that these selection pressures might play key roles in determining the structure of genes and organizing genetic information.

We also investigated the expression patterns and tissue specificity of PSGs and NSGs based on RNA-seq data. The expression levels of PSGs were lower than those of NSGs, and PSGs exhibited more pronounced tissue-specific expression patterns. The relationship between selective mode and codon usage bias was also analyzed. The Ka/Ks ratios were significantly negatively correlated with CAI, CBI, and Fop. PSGs exhibited lower CAI, CBI, and Fop values compared to NSGs, suggesting that codon usage bias was more pronounced for NSGs. The rate of missense mutation and codon usage bias was conserved among barley NSGs. We speculate that artificial selection during barley domestication affected gene expression profiles, including patterns of tissue-specific expression.

### PSGs have experienced more severe genetic bottlenecks than NSGs during the domestication of barley

The π of PSGs and NSGs was compared using the exome resequencing data to determine whether PSGs and NSGs have undergone genetic bottlenecks during barley domestication. Most crops experience a severe decrease in genetic diversity during domestication. For example, genetic diversity decreased by 52% from wild soybean to landrace soybean and by 25% from landrace soybean to an improved soybean cultivar [61]. An average reduction in π of approximately 20% was observed in maize landraces relative to their wild ancestors [62]. Genetic diversity decreased from 3.0 × 10^−3^ in wild emmer to 1.3 × 10^−3^ in hexaploid landraces for the A subgenome, and from 3.1 × 10^−3^ to 1.4 × 10^−3^ for the B subgenome [63]. π decreased by 27% in landrace barley relative to wild barley during domestication [48]. In our study, we observed an average reduction in π of 13.87% across all the PSGs and NSGs, which was slightly lower than that across the whole genome [48]. These inconsistent results might be explained by the different methods used to evaluate π levels. The decrease in π was estimated to be 27% at the whole-genome level based on the ‘window-pi’ method; however, the ‘site-pi’ method was used in our study given the asymmetry in the distributions of the PSGs and NSGs. The single-copy genes are largely housekeeping genes, which are relatively conserved and inherited linearly [64]. The decrease in the π of PSGs was lower than that of NSGs (17.00% *vs*. 13.41%), suggesting that intense artificial selection caused a more severe genetic bottleneck, particularly when selection acts strongly on the PSGs possessed by only a subset of the barley population.

### PSGs may play important roles in barley growth and development in barley

GO and KEGG analyses provided insight into the potential functions of barley PSGs. The GO terms, such as fertilization (GO:0009566), nitrogen compound metabolic process (GO:0006807), and floral organ development (GO:0048437), and the KEGG pathways, such as protein families: genetic information processing, barite hierarchies, ubiquitin system, and RNA polymerase were highly enriched. These results indicate that barley PSGs play key roles in diverse physiological and developmental processes.

Gene expression analyses enhance our understanding of the functions of genes. The spatiotemporal expression patterns of genes in multiple tissues/stages suggest that the identified PSGs may play a key role in the growth and development of barley. For example, *HORVU.MOREX.r2.3HG0191990* was differentially expressed in the grain. Its orthologous gene *NAC31* is essential for secondary wall biosynthesis in rice, mainly through a gibberellin-mediated DELLA-NAC signaling cascade [65, 66]. Another bHLH family gene, *HORVU.MOREX.r2.2HG0161050*, is highly expressed in the tillers, lodicules, lemmas, and embryos. Analysis of gene orthologs revealed the orthologous gene *EAT1*, which encodes a TF that is key for inducing programmed cell death in post-meiotic anther tapetum, the somatic nursery for pollen production [67, 68]. *HORVU.MOREX.r2.4HG0314480,* a PSG that arose during barley domestication, was highly expressed in the young inflorescences and senescing leaves. Its orthologous gene *DEP2* encodes a plant-specific protein without a known functional domain that is involved in panicle outgrowth and elongation [69]. Remarkably, the *PROG1* gene regulates architecture in wild rice, which was one of the most critical phenotypes during rice domestication. *PROG1* was functionally lost in cultivated rice through artificial selection. Its orthologous gene *HORVU.MOREX.r2.4HG0344630*, which was not expressed in any tissue/stage, was under positive selection during barley domestication, implying the convergent selection pattern that may have occurred in barley and rice [70, 71]. In sum, functional analysis of these candidate genes will further aid our knowledge of barley PSGs.

Haplotype construction and characterization provide insight into the differentiation of important genes and the processes underlying their evolution [72]. Haplotype networks reveal that levels of haplotype polymorphisms of these candidate PSGs in wild barley were high compared to those in landrace barley, suggesting that the initial effects of artificial selection during domestication involved promoting the retention of specific haplotypes and eliminating unfavorable haplotypes. The wild barley population possessed specific haplotypes that were absent in the domesticated population, which suggests that the genetic traits controlled by PSGs in domesticated barley could be enriched. Characterizing the haplotypes of PSGs will provide new insight into the functional divergence between cultivated and wild barley and will help establish associations between genetic variants and important agronomic traits, which would allow them to be used as molecular markers.

## Conclusions

This is the first study to conduct a comparative analysis of the PSGs and NSGs in barley. Our results suggest that artificial selection has been the dominant factor affecting the evolutionary rate, compactness, and expression of genes, as well as the genetic diversity in barley. PSGs associated with domestication and improvement of barley could be studied in future functional investigations and used as targets in barley breeding programs.

## Methods

### Data sources and identification of orthologous gene pairs in barley

The coding sequences (CDS), protein sequences, and generic feature format (gff) file of Morex V2 (*Hordeum vulgare*, referred to as the improved barley cultivar) were downloaded from the Leibniz Institute of Plant Genetics and Crop Plant Research (IPK) (https://doi.org/10.5447/ipk/2019/8). The genomic resources of B1K-04–12 (*Hordeum spontaneum*, referred to as wild barley) and HOR 10350 (*Hordeum vulgare*, referred to as landrace barley) were also available from the IPK database (https://webblast.ipk-gatersleben.de/downloads/barley_pangenome/). OrthoFinder v2.5.4 and OrthoMCL v2.0.9 were used to identify the orthologous gene pairs with default parameters [73, 74]. Syntenic analysis was performed using jcvi v1.1.18 (https://github.com/tanghaibao/jcvi) [75].

### Characterization of selection modes

The multiple sequence alignment was carried out using full-length proteins by Clustal v1.2.4 [76]. The PAL2NAL program (http://www.bork.embl.de/pal2nal/) was used to generate codon alignments. The Ka, Ks, and Ka/Ks values were calculated using codeml in Phylogenetic Analysis by Maximum Likelihood (PAML) v4.9 [29]. Orthologous gene pairs with Ks > 0.3 were eliminated prior to subsequent analyses due to possible saturation of synonymous substitutions [25]. We also discarded gene pairs with Ka = 0 or Ks = 0, which suggests that they could have experienced strong negative or positive selection, respectively [26].

### Analysis of gene structure and codon usage bias

An inhouse python script was developed to calculate the gene length, intron length, exon length, first exon length, and exon number for PSGs and NSGs based on the gff annotation files. To estimate codon usage bias, only protein sequences longer than 100 amino acids (300 bp CDS) were preserved for subsequent analyses. The GC content, CAI, CBI, and Fop were calculated using CodonW v1.4.4.

### Expression profiling analysis

A total of 73 RNA-seq samples from 12 tissues/stages were retrieved from the National Center for Biotechnology Information (NCBI) Sequence Reading Archive (SRA) database (PRJEB14349). The accession numbers and details are provided in Table S19. SRA format files were converted to FASTQ format using the parallel-fastq-dump package (https://github.com/rvalieris/parallel-fastq-dump). Quality control was performed using Trimmomatic v0.36 (http://www.usadellab.org/cms/index.php?page=trimmomatic) [77]. The high-confidence reads were aligned to the reference genome (Morex V2) using HISAT2 v 2.1.0 [78]. BAM files were sorted by coordinate using the sort function in SAMtools v1.3.1. StringTie v1.3.5 was used to calculate FPKM values based on the genomic annotation file [79]. The index τ was used to estimate the tissue specificity for each gene according to the formula:

$$\tau = \frac{{\sum }_{i}^{N} (1 - \frac{{x}_{i}}{{x}_{max}})}{N-1}$$

where N indicates the number of tissues, *x*_*i*_ indicates the mean value of FPKM in tissue *i*, and *x*_*max*_ is the maximum FPKM among all tissues [80, 81]. The τ values ranged from 0 to 1, with τ = 1 representing absolute specificity and τ = 0 representing equal expression in all tissues.

### Identification of TFs and functional annotation of PSGs

TF identification was performed using online tools in the Plant Transcription Factor Database (PlantTFDB v5.0, http://planttfdb.gao-lab.org/index.php). GO and KEGG annotations were performed using eggnog-mapper v2.1.7 online database (http://eggnog-mapper.embl.de). Enrichment analysis was carried out using the TBtools v1.098726. GO terms and KEGG pathways with *P* value < 0.05 were considered statistically significant. The enriched GO terms were visualized using WocEA v1.0 [82].

### Nucleotide variant calling and population genetic analysis

The barley genomic exome-captured sequencing datasets, including 85 wild accessions and 133 landrace accessions, were downloaded from the NCBI SRA database (PRJEB8044/ERP009079) [48]. GenBank accession numbers and voucher information are listed in Table S20. Raw sequence reads were preprocessed using Trimmomatic v 0.36 (http://www.usadellab.org/cms/index.php?page=trimmomatic) [77]. The reference genome index of barley Morex V2 was constructed using the index function of BWA v0.7.13r1126. The high-quality reads were mapped to the reference genome using the BWA-MEM algorithm. Picard tools (https://broadinstitute.github.io/picard/) was used to sort BAM files and mark possible PCR duplicates. Variant calling was performed using GATK v4.2.5.0 HaplotypeCaller (https://software.broadinstitute.org/gatk/). The SNPs generated were filtered using the following criteria: Quality by Depth (QD) < 2.0; Mapping Quality (MQ) < 40.0; Fisher Strand (FS) > 60.0, Strand Odds Ratio (SOR) > 3.0; Mapping Quality Rank Sum (MQRankSum) < –12.5; and Read Position Rank Sum (ReadPosRankSum) < –8.0. Functional variant annotation was carried out using the SnpEff v4.3 tool (https://pcingola.github.io/SnpEff/). SNPs with minor allele frequency (MAF) < 0.05 were discarded. PCA was carried out using the smartpca package of EIGENSOFT v6.1.4. The significance of different components was determined using the Tracy-Widom statistic test [83]. A neighbor-joining (NJ) phylogenetic tree was constructed using TreeBeST v1.9.2 with 1,000 bootstrap replicates, and FigTree v1.4.4 was used to visualize the tree (http://tree.bio.ed.ac.uk/software/figtree/). Population structure was inferred by ADMIXTURE v1.3.0 with a K ranging from 2 to 4 [84]. π and *F*_*ST*_ values were calculated using VCFtools v0.1.17 [85].

### Orthologous gene identification and haplotype analysis

To identify the possible functions of the candidate genes, BLAST v2.12.0 was used to conduct a search against the rice protein database with a cut-off of 75% identity and an E-value of 1e–5. Data on the expression profile, gene ontology, biological function, and phenotype were obtained from the China Rice Data Center (https://www.ricedata.cn/gene/). The number of haplotypes was calculated in DnaSP v6.12.03, and haplotype networks were constructed using PopART v1.7 with the median-joining method [86–88]. The intron–exon gene structure and SNP locations were visualized using the online tools in Gene Structure Display Server (GSDS) v2.0 (http://gsds.gao-lab.org/) [89].

### Plotting and statistical tests

The frequency distribution, scatter, box, violin, density, and stacked graphs were generated using the ggplot2 package in R. Figure panels were assembled using the cowplot package. The chromosomal distributions of the Ka, Ks, and Ka/Ks values were displayed using the RIdeogram package. The heat maps were visualized using the pheatmap package, and the expression profile was generated with the log2 transformed FPKM values. The corrplot package was used to make the correlation heat maps. One-sided Mann–Whitney *U*-test, one-sided Fisher’s exact test, and Spearman’s rank correlation test were performed using the base R package wilcox.test, chisq.test, and cor.test functions, respectively. Levels of statistical significance were set at * for *P* < 0.05, ** for *P* < 0.01, and *** for *P* < 0.001.

## Supplementary Information

**Additional file 1: Figure S1.** Syntenic relationships of the orthologs among wild barley, landrace, and improved cultivar.

**Additional file 2: Figure S2. **Frequency distributions and correlation analysis of Ka, Ks and Ka/Ks between landrace and improved cultivar. **a-c** The frequency shows of Ka, Ks and Ka/Ks, respectively. **d** The correlation between Ks (x-axis) and Ka. **e** The correlation between Ks (x-axis) and Ka/Ks. **f** The correlation between Ka (x-axis) and Ka/Ks.

**Additional file 3: Figure S3.** Correlations among substitution rates, gene features, codon usage bias and expression patterns between landrace and improved cultivar. Upper Right: the size of the circle represents the magnitude of the correlation coefficient, red reveals positive correlation, and blue reveals negative correlation. One asterisk (*), double asterisk (**) and triple asterisk (***) indicate 0.05, 0.01 and 0.001 significant difference level, respectively. Bottom Left: correlation coefficients are presented as Spearman’s rank correlation test ρ.

**Additional file 4: Figure S4.** Distributions of Ka, Ks, and Ka/Ks values alongside the chromosome with a bin size of 50 consecutive orthologs. **a-c** The genomic distributions of Ka, Ks and Ka/Ks values alongside chromosome blocks between wild barleyand landrace. **d-e** The genomic distributions of Ka, Ks and Ka/Ks values alongside chromosome blocks between landrace and improved cultivar.

**Additional file 5: Figure S5. **Distributions of Ka, Ks, and Ka/Ks values between landrace and improved cultivar alongside the chromosome.

**Additional file 6: Figure S6. **Frequency distributions of genomic features between PSGs and NSGs during the process of barley domestication. **a-l** The density plots display Ka, Ks, Ka/Ks, gene length, intron length, exon length, first exon length, exon number, GC content, CAI, CBI and FOP between two selections. Blue and purple lines represent positively and negatively selected genes, respectively.

**Additional file 7: Figure S7.** Frequency distributions of genomic features between PSGs and NSGs during the process of barley improvement. **a-n** The plots display of Ka, Ks, Ka/Ks, gene length, intron length, exon length, first exon length, exon number, GC content, CAI, CBI, FOP, expression level (FPKM) and tissue specificity (τ) between two selections. Blue and purple lines represent positively and negatively selected genes, respectively.

**Additional file 8: Figure S8. **Comparisons of genomic features between PSGs and NSGs during the process of barley improvement. The line in the box is the median value, and the lines at the bottom and top of each box are the first (lower) and third (higher) quartiles. Violin plots represent the density of gene numbers. **a-n **The plots display of Ka, Ks, Ka/Ks, gene length, intron length, exon length, first exon length, exon number, GC content, CAI, CBI, FOP, expression level (FPKM) and tissue specificity (τ) between two selections. Blue and purple boxes represent positively and negatively selected genes, respectively.

**Additional file 9: Figure S9.** Identification of TFs and functional enrichment analysis of PSGs between landrace and improved cultivar. **a** Frequency distributions of transcription factors. Blue and purple columns represent positively and negatively selective genes, respectively. **b **Word clouds of GO terms of PSGs. The larger word of the term indicates gene numbers that were more enriched. Terms with *P *< 0.05 were selected.

**Additional file 10: Figure S10.** Distributions of Gene Ontology terms between wild barley and landrace. Yellow, green, and pink columns represent biological process, cellular component, and molecular function, respectively. Terms with *P *< 0.05 were selected.

**Additional file 11: Figure S11.** Distributions of Gene Ontology terms between landrace and improved cultivar barley. Yellow, green and pink columns represent biological process, cellular component and molecular function, respectively. Terms with *P* < 0.05 were selected.

**Additional file 12: Figure S12.** Population structure based on PSG-related SNPs. **a** Principal component analysis PC1 *vs.* PC2. **b** Principal component analysis PC1 *vs.* PC3. **c** The NJ phylogenetic tree. **d **Population structure with K ranging from 2 to 4.

**Additional file 13: Figure S13.** Population structure based on NSG-related SNPs. **a** Principal component analysis PC1 *vs.* PC2. **b** Principal component analysis PC1 *vs.* PC3. **c** The NJ phylogenetic tree. **d **Population structure with K ranging from 2 to 4.

**Additionalfile 14: Table S1.** Statistics of orthologs, syntenic gene pairs, and syntenic blocks. **Table S2.** The Ka, Ks, and Ka/Ks values for 9,176 and 6,483 single-copy orthologous genes between wild barley and landrace, and between landrace, and improved cultivar, respectively. **Table S3.** Correlation analysis of substitution rate, gene feature, and codon usage bias.(The Ka, Ks, and Ka/Ks values were calculated between wild barley and landrace). **Table S4.** Correlation analysis of substitution rate, gene feature, codon usage bias and expression pattern. (The Ka, Ks, and Ka/Ks values were calculated between landrace and improved barley). **Table S5.** Distributions of Ka/Ks values between wild barley and landrace, and between landrace and improved cultivar. **Table S6. **Comparisons of evolutionary rate, gene property, codon usage bias between PSGs and NSGs in barley. **Table S7.** Comparisons of expression patterns between PSGs and NSGs in barley. **Table S8.** Statistics of codon usage bias indicators. **Table S9.** Distributions of transcription factor gene family for different orthologous groups. **Table S10.** Statistics of the 49 representative transcription factor gene families. **Table S11.** The detail information of positively selected transcription factors. **Table S12.** GO enrichment analysis of PSGs. **Table S13.** KEGG pathway enrichment analysis of PSGs. **Table S14.** Distributions of PSG-related and NSG-related SNPs. **Table S15.** Tracy-Widom test for the first five eigenvectors in the PCA. **Table S16.** Nucleotide diversity (π) analysis between PSGs and NSGs. **Table S17.**Comparisons of *F*_*ST*_ values between PSGs and NSGs within different genomic regions. **Table S18.** SNPs distributions, nucleotide diversities, haplotypes, and expression patterns of candidate genes. **Table S19. **Accession numbers and sample information of the RNA-seq data used in this study. **Table S20.** Accession numbers and information of the 85 wild barley and 133 landrace accessions.

## Data Availability

Data pertaining to the study have been included in the article, and further inquiries can be directed to the corresponding authors. The sequences of improved cultivar barley Morex V2 (*Hordeum vulgare*) is available in the Leibniz Institute of Plant Genetics and Crop Plant Research (IPK) (https://doi.org/10.5447/ipk/2019/8). The sequences of wild barley B1K-04–12 (*Hordeum spontaneum*) and landrace barley HOR 10350 (*Hordeum vulgare*) are also available in the IPK database (https://webblast.ipk-gatersleben.de/downloads/barley_pangenome/). The gene expression data and exome-capture resequencing data were downloaded from the NCBI database (http://www.ncbi.nlm.nih.gov/geo/) under BioProject accession number PRJEB14349 and PRJEB8044/ERP009079.

## Abbreviations

BP: Biological Process
CAI: Codon Adaptation Index
CBI: Codon Bias Index
CC: Cellular Component
CDS: Coding Sequences
FDR: False Discovery Rate
Fop: Frequency of Optimal Codons
FPKM: Fragments Per Kilobase of Exon Per Million Fragments Mapped
FS: Strand Filter
*F*_*ST*_: Wright’s F-statistic
GFF: Generic Feature Format
GO: Gene Ontology
GSDS: Gene Structure Display Sever
IPK: Leibniz Institute of Plant Genetics and Crop Plant Research
Ka: Nonsynonymous substitution
KEGG: Kyoto Encyclopedia of Genes and Genomes
Ks: Synonymous substitution
MAF: Minor Allele Frequency
MF: Molecular function
MQ: Mapping Quality
MQRankSum: Mapping Quality Rank Sum
NJ: Neighbor-Joining
NSG: Negatively Selected Gene
PAML: Phylogenetic Analysis by Maximum Likelihood
PCA: Principal Component Analysis
PlantTFDB: Plant Transcription Factor Database
PSG: Positively Selected Gene
QD: Quality by Depth
ReadPosRankSum: Read Position Rank Sum
SNP: Single Nucleotide Polymorphism
SOR: Strand Odds Ratio
SRA: Sequence Read Archive
TF: Transcription Factor
π: Nucleotide diversity
