# Supplementary figures and images for: Genomic insights into positive selection during barley domestication
Source: BMC Plant Biol. 2022 Jun 1;22:267. doi: 10.1186/s12870-022-03655-0 (PMC9158214; doi:10.1186/s12870-022-03655-0)

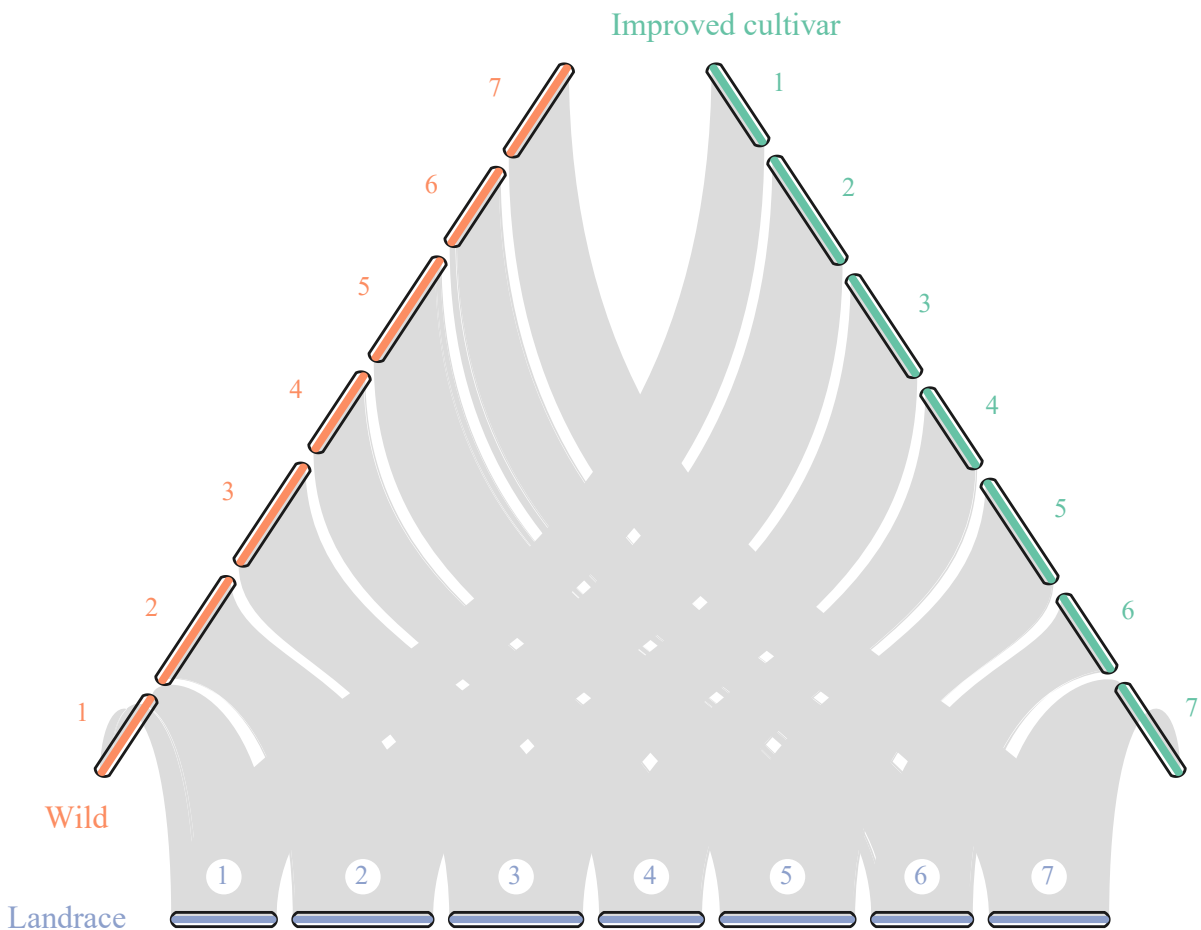

Supplement: Supplementary file 1 — Additional file 1: Figure S1. Syntenic relationships of the orthologs among wild barley, landrace, and improved cultivar. [file 12870_2022_3655_MOESM1_ESM.pdf]

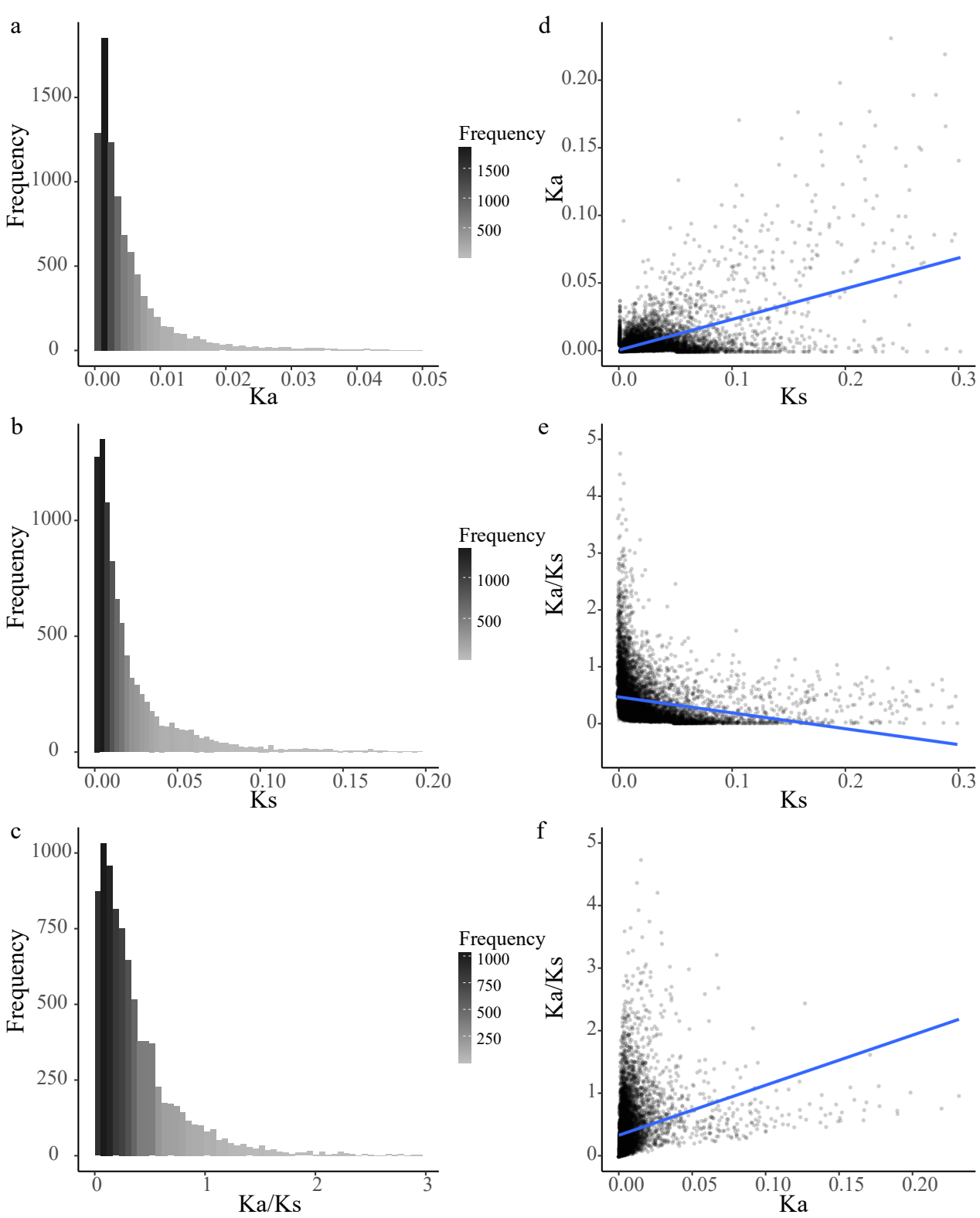

Supplement: Supplementary file 2 — Additional file 2: Figure S2. Frequency distributions and correlation analysis of Ka, Ks and Ka/Ks between landrace and improved cultivar. a-c The frequency shows of Ka, Ks and Ka/Ks, respectively. d The correlation between Ks (x-axis) and Ka. e The correlation between Ks (x-axis) and Ka/Ks. f The correlation between Ka (x-axis) and Ka/Ks. [file 12870_2022_3655_MOESM2_ESM.pdf]

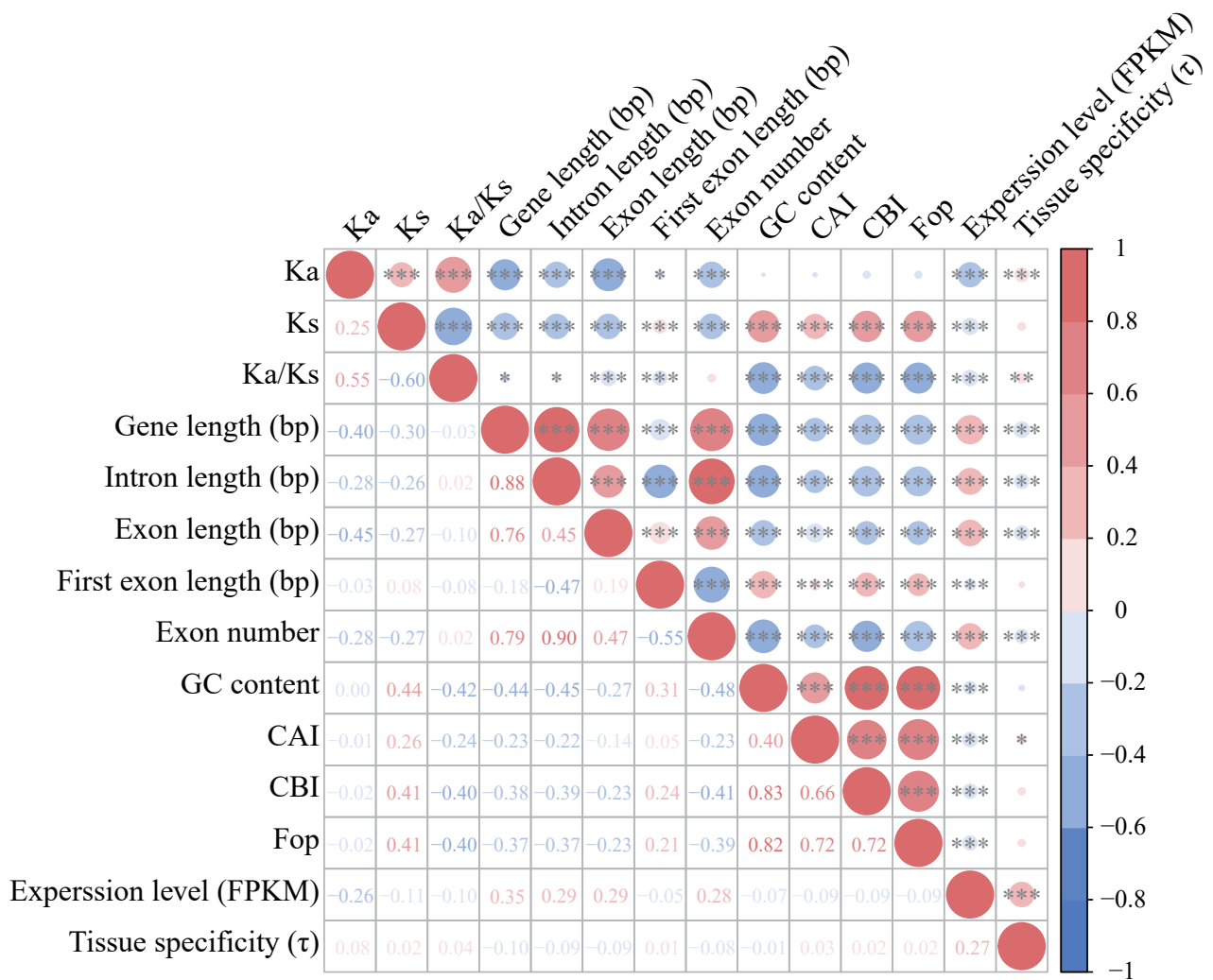

Supplement: Supplementary file 3 — Additional file 3: Figure S3. Correlations among substitution rates, gene features, codon usage bias and expression patterns between landrace and improved cultivar. Upper Right: the size of the circle represents the magnitude of the correlation coefficient, red reveals positive correlation, and blue reveals negative correlation. One asterisk (*), double asterisk (**) and triple asterisk (***) indicate 0.05, 0.01 and 0.001 significant difference level, respectively. Bottom Left: correlation coefficients are presented as Spearman’s rank correlation test ρ. [file 12870_2022_3655_MOESM3_ESM.pdf]

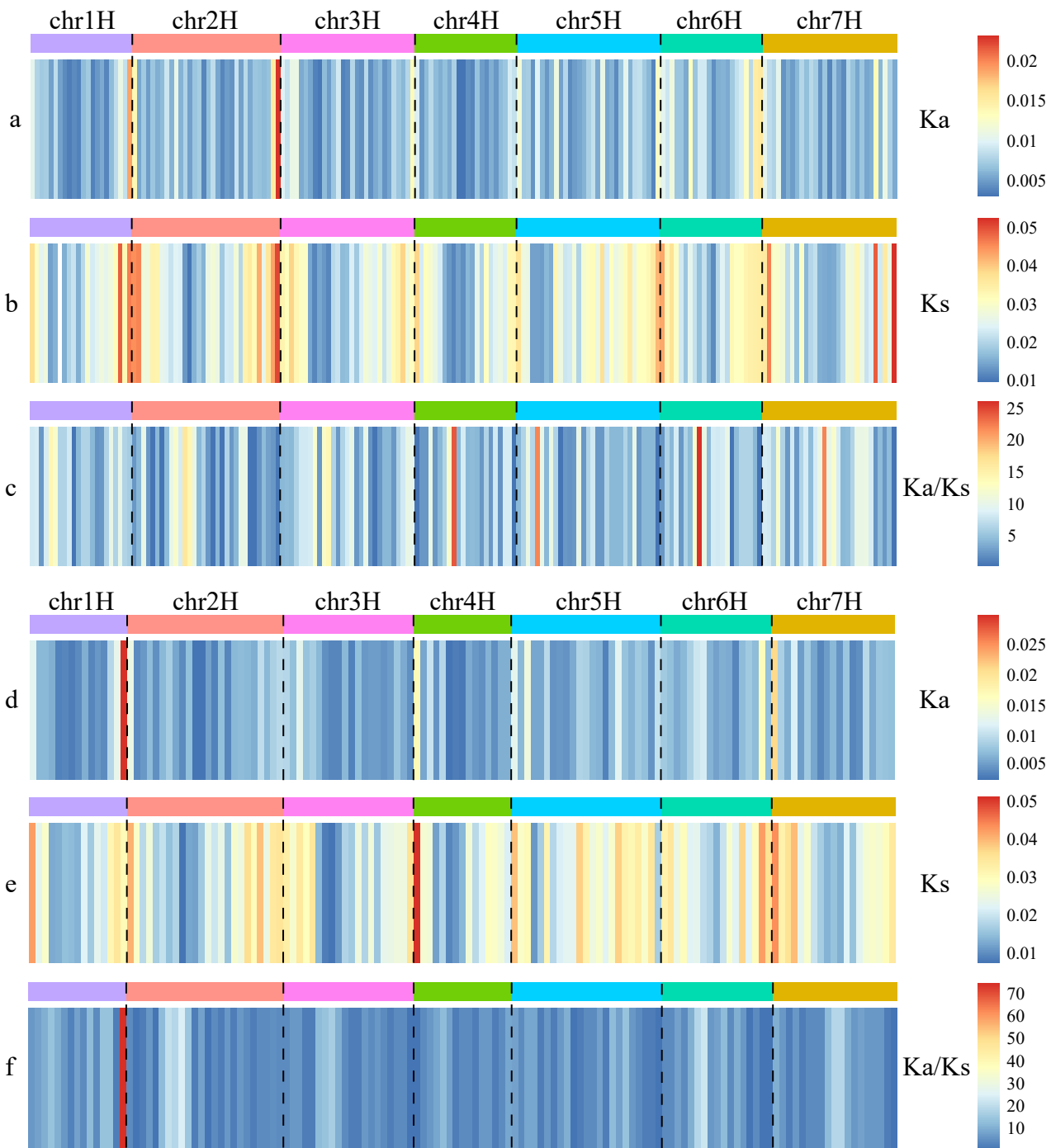

Supplement: Supplementary file 4 — Additional file 4: Figure S4. Distributions of Ka, Ks, and Ka/Ks values alongside the chromosome with a bin size of 50 consecutive orthologs. a-c The genomic distributions of Ka, Ks and Ka/Ks values alongside chromosome blocks between wild barleyand landrace. d-e The genomic distributions of Ka, Ks and Ka/Ks values alongside chromosome blocks between landrace and improved cultivar. [file 12870_2022_3655_MOESM4_ESM.pdf]

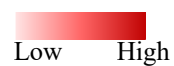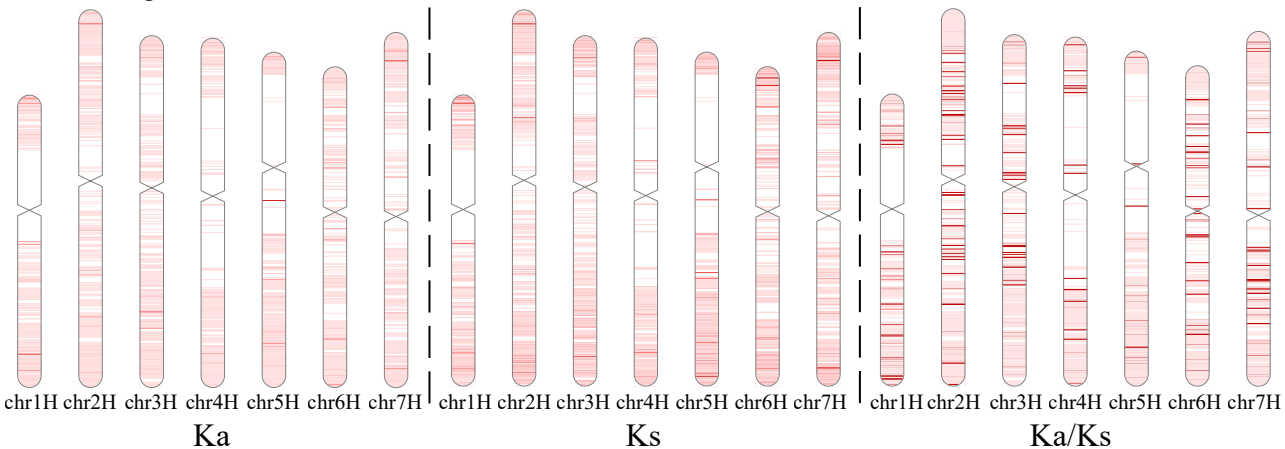

Supplement: Supplementary file 5 — Additional file 5: Figure S5. Distributions of Ka, Ks, and Ka/Ks values between landrace and improved cultivar alongside the chromosome. [file 12870_2022_3655_MOESM5_ESM.pdf]

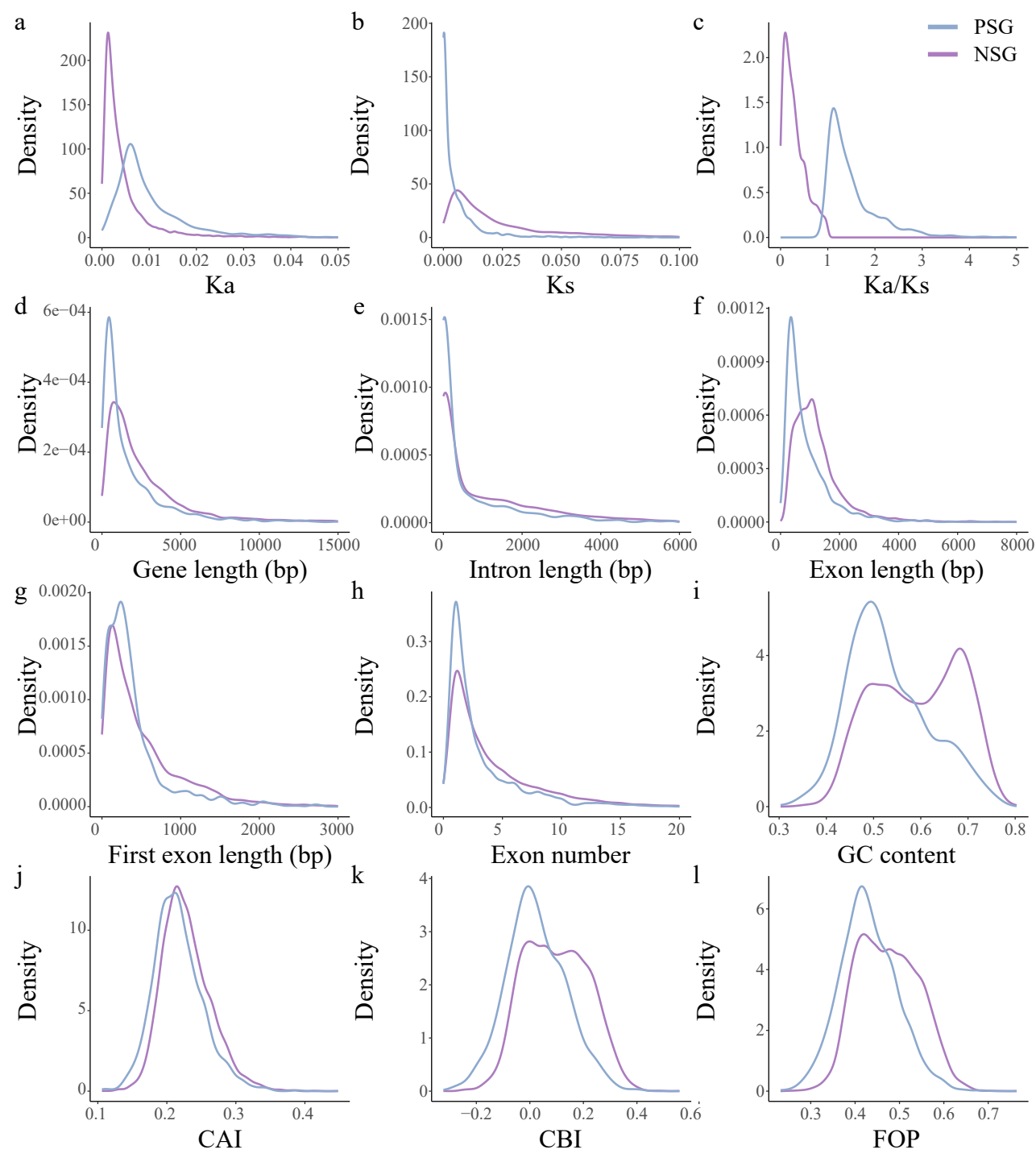

Supplement: Supplementary file 6 — Additional file 6: Figure S6. Frequency distributions of genomic features between PSGs and NSGs during the process of barley domestication. a-l The density plots display Ka, Ks, Ka/Ks, gene length, intron length, exon length, first exon length, exon number, GC content, CAI, CBI and FOP between two selections. Blue and purple lines represent positively and negatively selected genes, respectively. [file 12870_2022_3655_MOESM6_ESM.pdf]

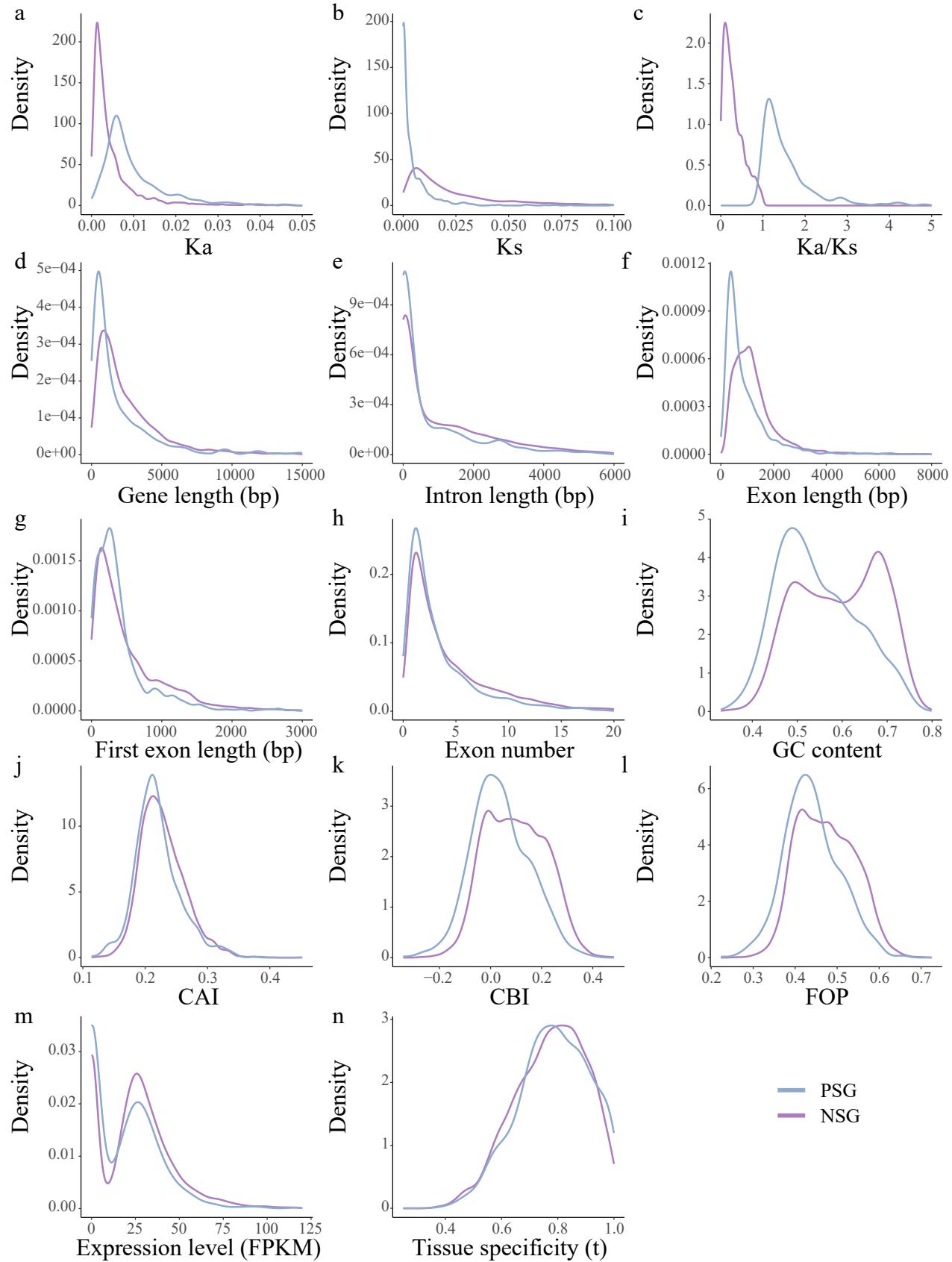

Supplement: Supplementary file 7 — Additional file 7: Figure S7. Frequency distributions of genomic features between PSGs and NSGs during the process of barley improvement. a-n The plots display of Ka, Ks, Ka/Ks, gene length, intron length, exon length, first exon length, exon number, GC content, CAI, CBI, FOP, expression level (FPKM) and tissue specificity (τ) between two selections. Blue and purple lines represent positively and negatively selected genes, respectively. [file 12870_2022_3655_MOESM7_ESM.pdf]

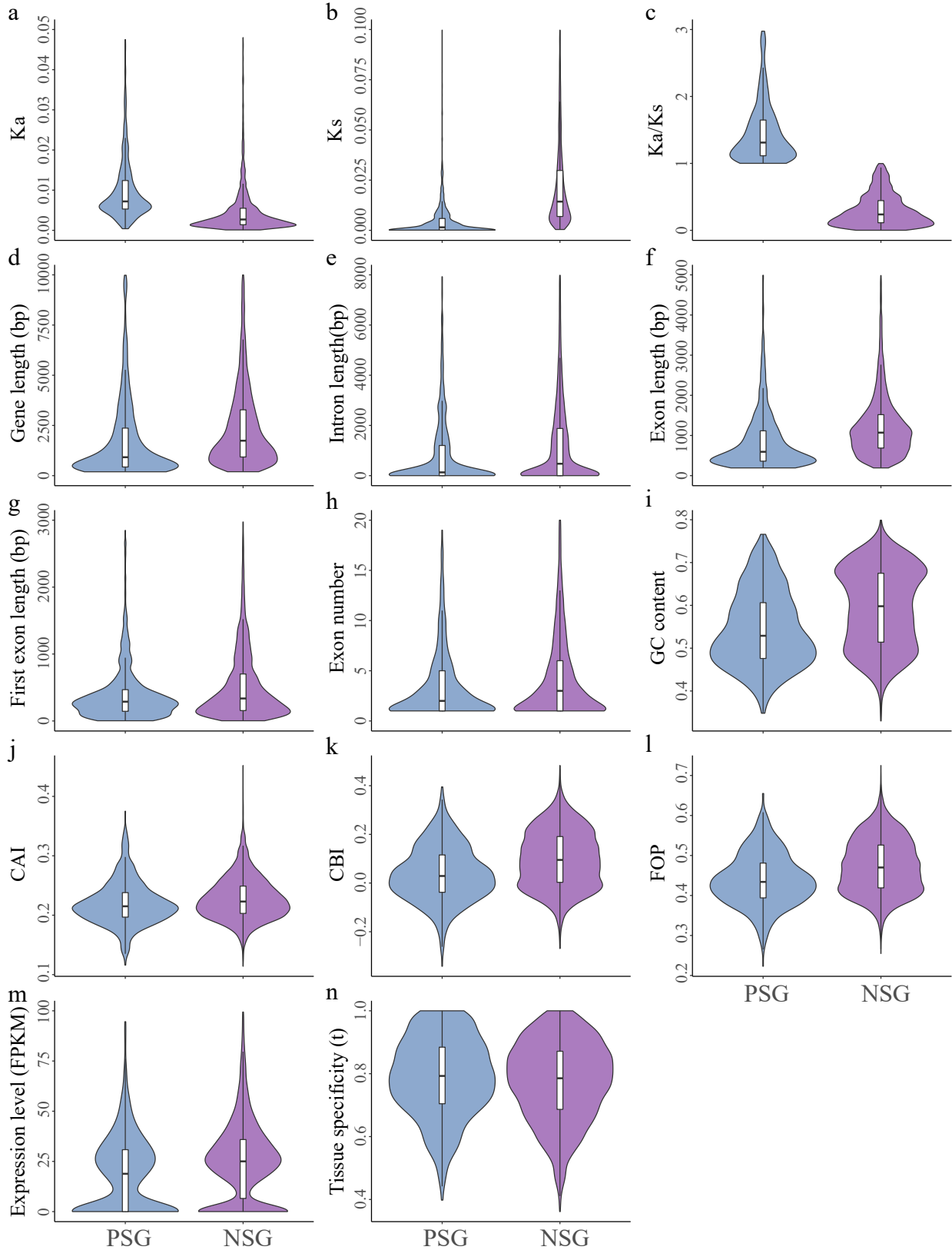

Supplement: Supplementary file 8 — Additional file 8: Figure S8. Comparisons of genomic features between PSGs and NSGs during the process of barley improvement. The line in the box is the median value, and the lines at the bottom and top of each box are the first (lower) and third (higher) quartiles. Violin plots represent the density of gene numbers. a-n The plots display of Ka, Ks, Ka/Ks, gene length, intron length, exon length, first exon length, exon number, GC content, CAI, CBI, FOP, expression level (FPKM) and tissue specificity (τ) between two selections. Blue and purple boxes represent positively and negatively selected genes, respectively. [file 12870_2022_3655_MOESM8_ESM.pdf]

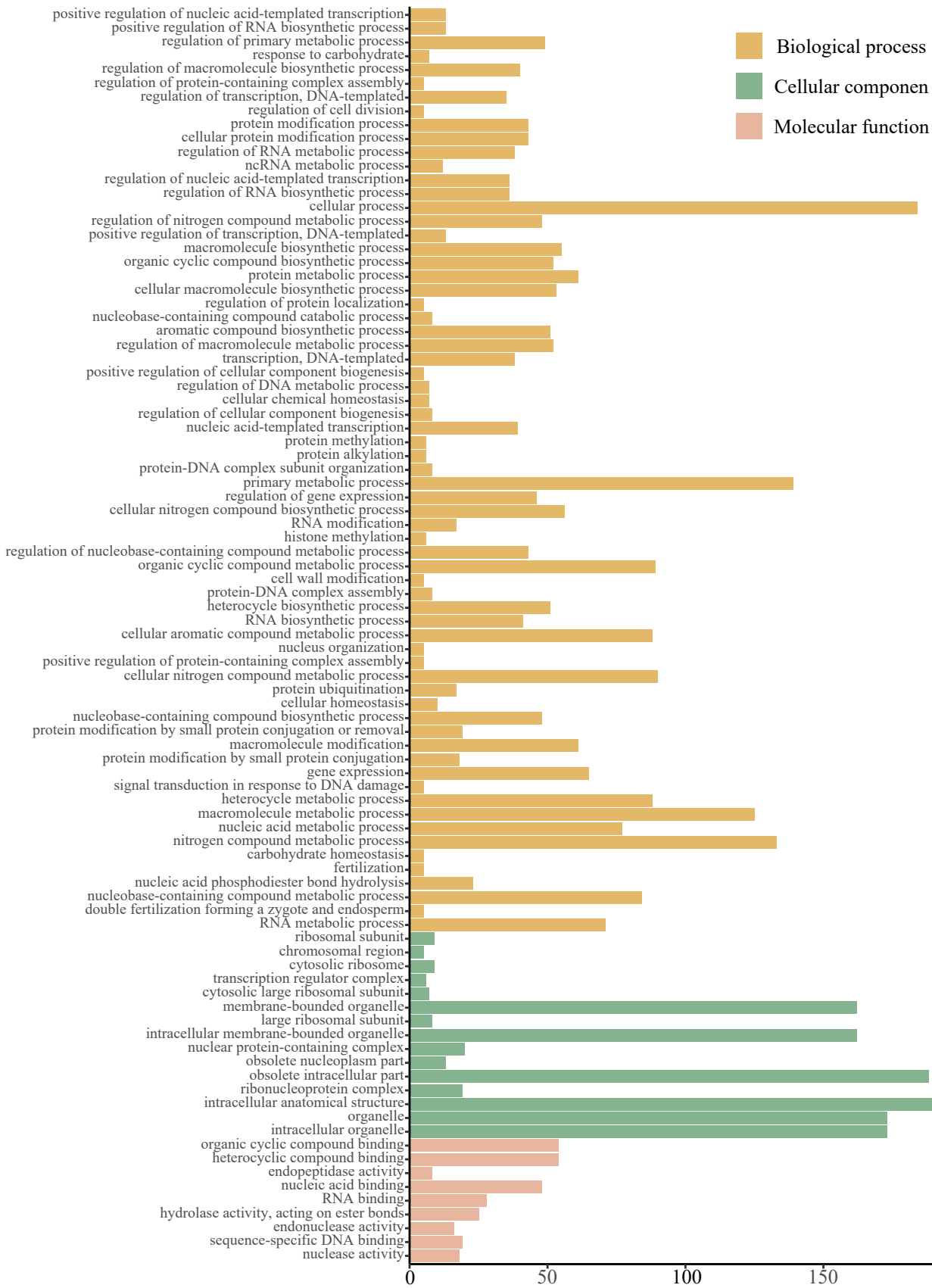

Supplement: Supplementary file 10 — Additional file 10: Figure S10. Distributions of Gene Ontology terms between wild barley and landrace. Yellow, green, and pink columns represent biological process, cellular component, and molecular function, respectively. Terms with P < 0.05 were selected. [file 12870_2022_3655_MOESM10_ESM.pdf]

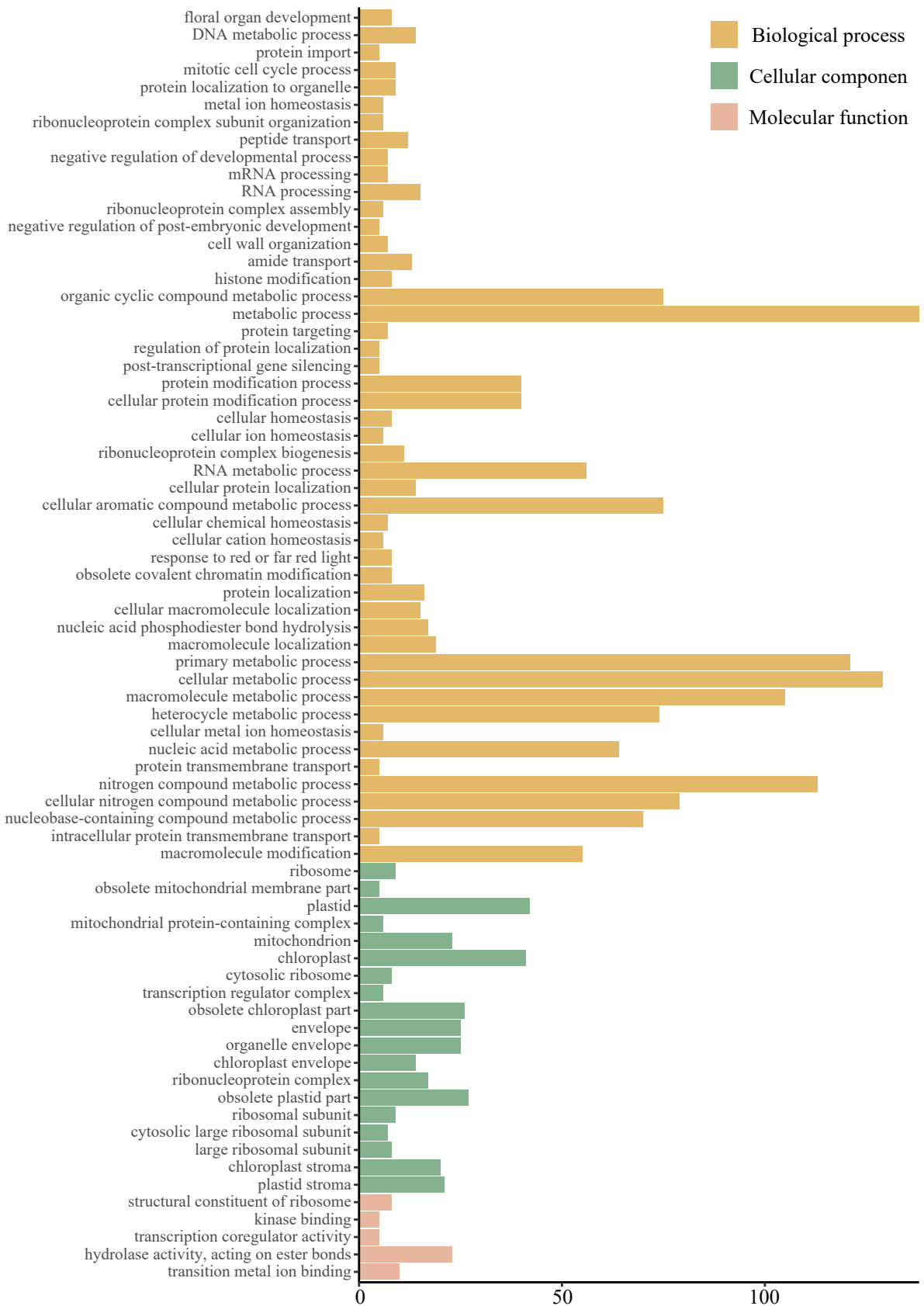

Supplement: Supplementary file 11 — Additional file 11: Figure S11. Distributions of Gene Ontology terms between landrace and improved cultivar barley. Yellow, green and pink columns represent biological process, cellular component and molecular function, respectively. Terms with P < 0.05 were selected. [file 12870_2022_3655_MOESM11_ESM.pdf]

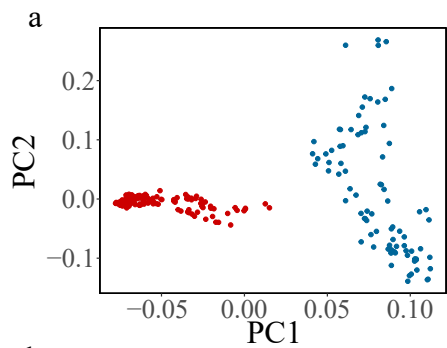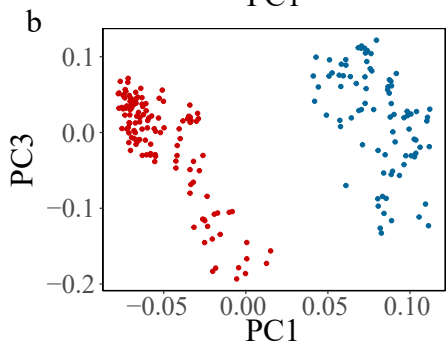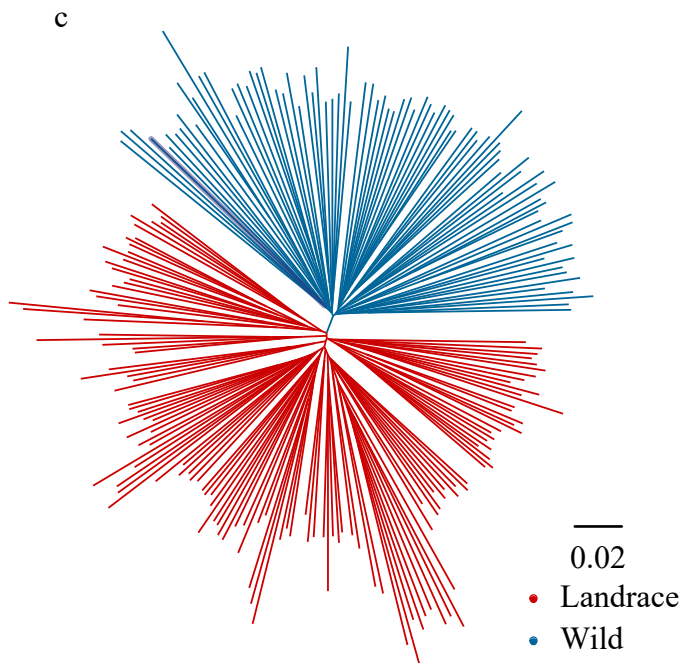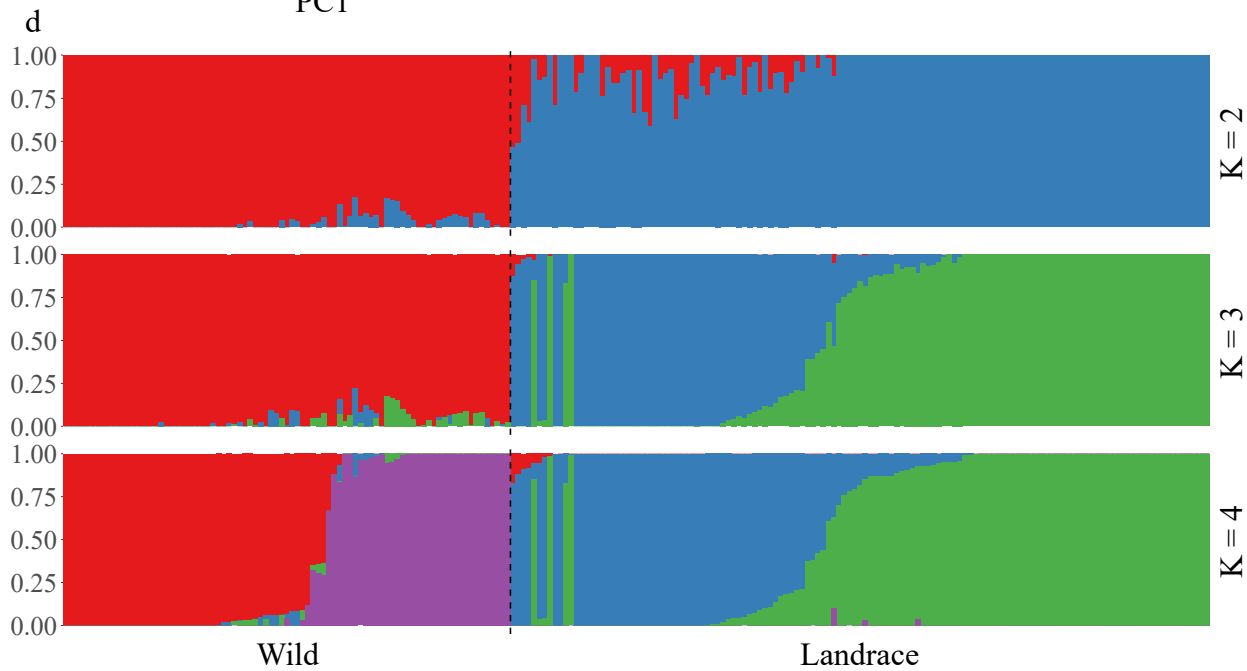

Supplement: Supplementary file 12 — Additional file 12: Figure S12. Population structure based on PSG-related SNPs. a Principal component analysis PC1 vs. PC2. b Principal component analysis PC1 vs. PC3. c The NJ phylogenetic tree. d Population structure with K ranging from 2 to 4. [file 12870_2022_3655_MOESM12_ESM.pdf]

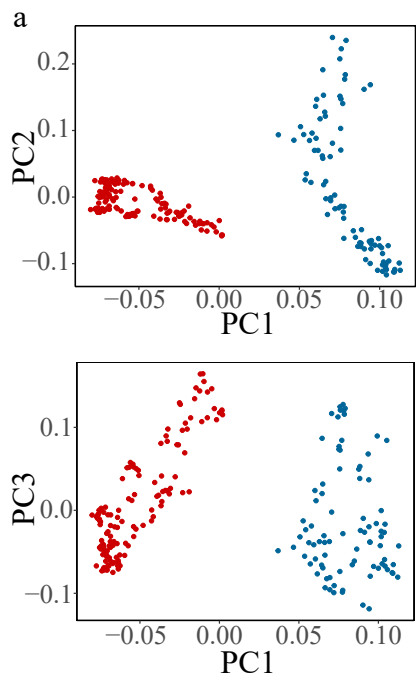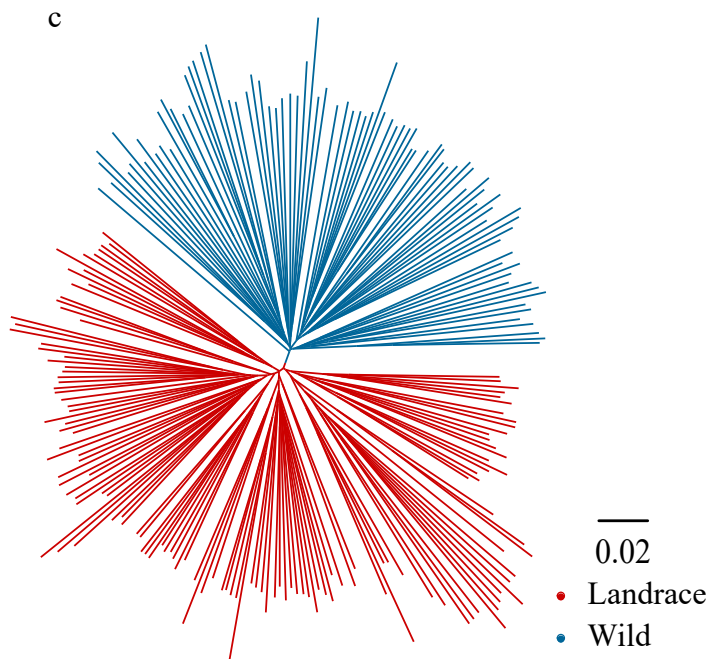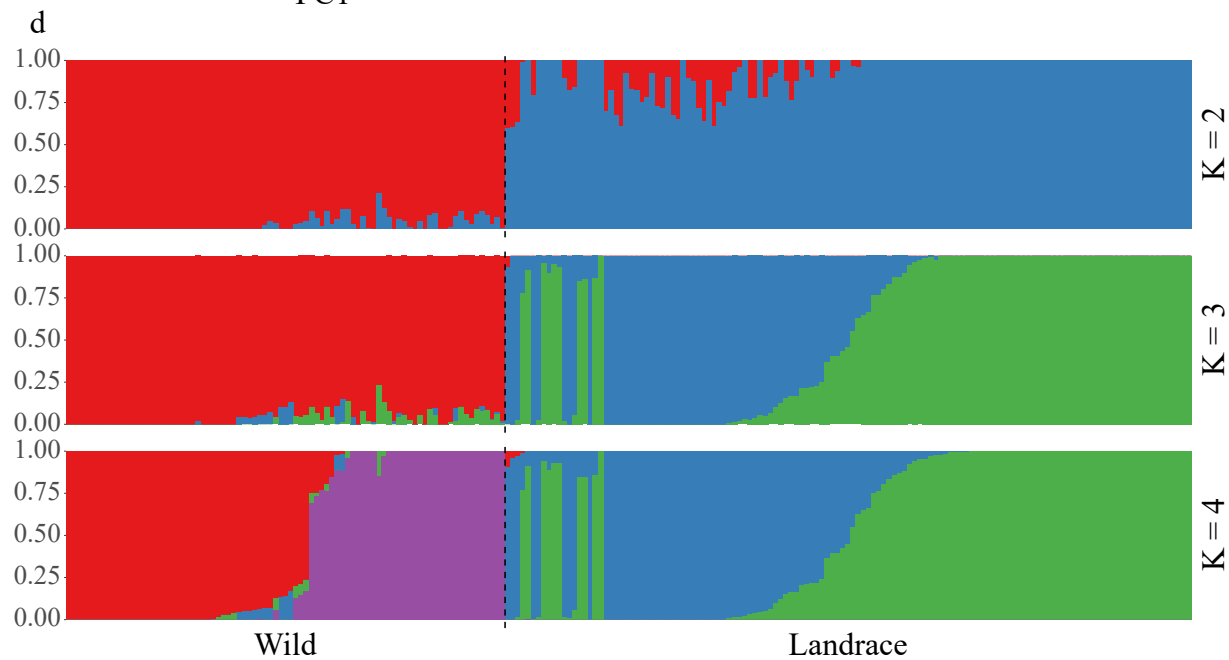

Supplement: Supplementary file 13 — Additional file 13: Figure S13. Population structure based on NSG-related SNPs. a Principal component analysis PC1 vs. PC2. b Principal component analysis PC1 vs. PC3. c The NJ phylogenetic tree. d Population structure with K ranging from 2 to 4. [file 12870_2022_3655_MOESM13_ESM.pdf]
