# Supplementary material for: Genomic insights into positive selection during barley domestication
Source: BMC Plant Biol. 2022 Jun 1;22:267. doi: 10.1186/s12870-022-03655-0 (PMC9158214; doi:10.1186/s12870-022-03655-0)
Supplement: Supplementary file 9 — Additional file 9: Figure S9. Identification of TFs and functional enrichment analysis of PSGs between landrace and improved cultivar. a Frequency distributions of transcription factors. Blue and purple columns represent positively and negatively selective genes, respectively. b Word clouds of GO terms of PSGs. The larger word of the term indicates gene numbers that were more enriched. Terms with P < 0.05 were selected. [file 12870_2022_3655_MOESM9_ESM.pdf]

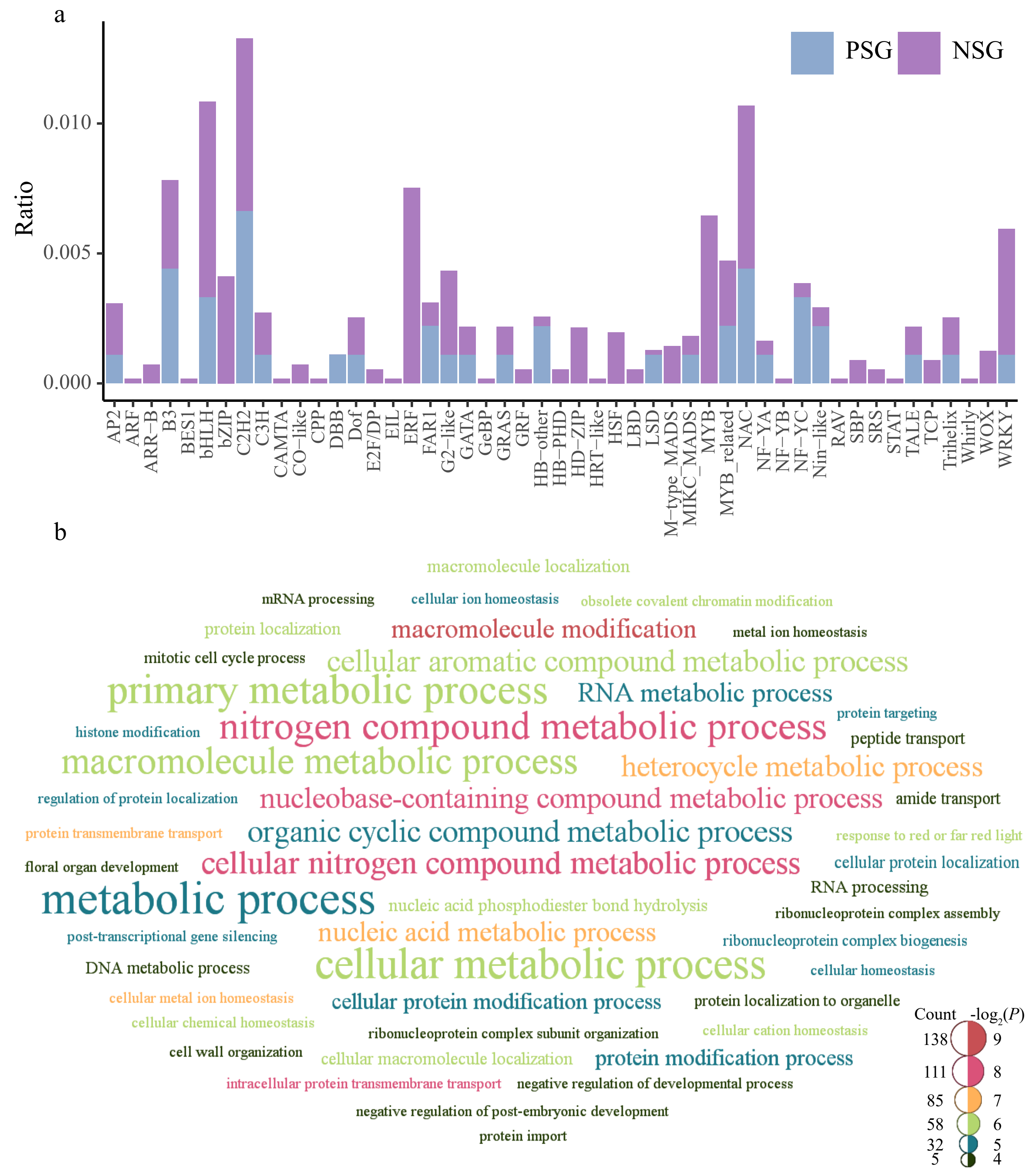
a
PSG
NSG
Ratio
0.010
0.005
0.000
AP2
ARF
ARR−B
B3
BES1
bHLH
bZIP
C2H2
C3H
CAMTA
CO−like
CPP
DBB
Dof
E2F/DP
EIL
ERF
FAR1
G2−like
GATA
GeBP
GRAS
GRF
HB−other
HB−PHD
HD−ZIP
HRT−like
HSF
LBD
LSD
M−type_MADS
MIKC_MADS
MYB
MYB_related
NAC
NF−YA
NF−YB
NF−YC
Nin−like
RAV
SBP
SRS
STAT
TALE
TCP
Trihelix
Whirly
WOX
WRKY
b
macromolecule localization
mRNA processing
cellular ion homeostasis
obsolete covalent chromatin modification
protein localization
macromolecule modification
metal ion homeostasis
mitotic cell cycle process
cellular aromatic compound metabolic process
primary metabolic process
RNA metabolic process
protein targeting
histone modification
nitrogen compound metabolic process
peptide transport
macromolecule metabolic process
heterocycle metabolic process
regulation of protein localization
nucleobase-containing compound metabolic process
amide transport
protein transmembrane transport
organic cyclic compound metabolic process
response to red or far red light
floral organ development
cellular nitrogen compound metabolic process
cellular protein localization
RNA processing
metabolic process
nucleic acid phosphodiester bond hydrolysis
ribonucleoprotein complex assembly
post-transcriptional gene silencing
nucleic acid metabolic process
ribonucleoprotein complex biogenesis
DNA metabolic process
cellular metabolic process
cellular homeostasis
cellular metal ion homeostasis
cellular protein modification process
protein localization to organelle
cellular chemical homeostasis
ribonucleoprotein complex subunit organization
cellular cation homeostasis
cell wall organization
cellular macromolecule localization
protein modification process
intracellular protein transmembrane transport
negative regulation of developmental process
negative regulation of post-embryonic development
protein import
Count
-log2(P)
138
111
85
58
32
5
9
8
7
6
5
4
